# Development and Evaluation of a Retrieval-Augmented Large Language Model Framework for Ophthalmology

**DOI:** 10.1001/jamaophthalmol.2024.2513

**Published:** 2024-07-18

**Authors:** Ming-Jie Luo, Jianyu Pang, Shaowei Bi, Yunxi Lai, Jiaman Zhao, Yuanrui Shang, Tingxin Cui, Yahan Yang, Zhenzhe Lin, Lanqin Zhao, Xiaohang Wu, Duoru Lin, Jingjing Chen, Haotian Lin

**Affiliations:** 1 State Key Laboratory of Ophthalmology, Zhongshan Ophthalmic Center, Sun Yat-sen University, Guangdong Provincial Key Laboratory of Ophthalmology and Visual Science, Guangdong Provincial Clinical Research Center for Ocular Diseases, Guangzhou, China; 2 The Second Affiliated Hospital of Xi’an Jiaotong University, Xi’an, China; 3 Center for Precision Medicine and Department of Genetics and Biomedical Informatics, Zhongshan School of Medicine, Sun Yat-sen University, Guangzhou, Guangdong, China; 4 Hainan Eye Hospital and Key Laboratory of Ophthalmology, Zhongshan Ophthalmic Center, Sun Yat-sen University, Haikou, China

## Abstract

**Importance:**

Although augmenting large language models (LLMs) with knowledge bases may improve medical domain–specific performance, practical methods are needed for local implementation of LLMs that address privacy concerns and enhance accessibility for health care professionals.

**Objective:**

To develop an accurate, cost-effective local implementation of an LLM to mitigate privacy concerns and support their practical deployment in health care settings.

**Design, Setting, and Participants:**

ChatZOC (Sun Yat-Sen University Zhongshan Ophthalmology Center), a retrieval-augmented LLM framework, was developed by enhancing a baseline LLM with a comprehensive ophthalmic dataset and evaluation framework (CODE), which includes over 30 000 pieces of ophthalmic knowledge. This LLM was benchmarked against 10 representative LLMs, including GPT-4 and GPT-3.5 Turbo (OpenAI), across 300 clinical questions in ophthalmology. The evaluation, involving a panel of medical experts and biomedical researchers, focused on accuracy, utility, and safety. A double-masked approach was used to try to minimize bias assessment across all models. The study used a comprehensive knowledge base derived from ophthalmic clinical practice, without directly involving clinical patients.

**Exposures:**

LLM response to clinical questions.

**Main Outcomes and Measures:**

Accuracy, utility, and safety of LLMs in responding to clinical questions.

**Results:**

The baseline model achieved a human ranking score of 0.48. The retrieval-augmented LLM had a score of 0.60, a difference of 0.12 (95% CI, 0.02-0.22; *P* = .02) from baseline and not different from GPT-4 with a score of 0.61 (difference = 0.01; 95% CI, −0.11 to 0.13; *P* = .89). For scientific consensus, the retrieval-augmented LLM was 84.0% compared with the baseline model of 46.5% (difference = 37.5%; 95% CI, 29.0%-46.0%; *P* < .001) and not different from GPT-4 with a value of 79.2% (difference = 4.8%; 95% CI, −0.3% to 10.0%; *P* = .06).

**Conclusions and Relevance:**

Results of this quality improvement study suggest that the integration of high-quality knowledge bases improved the LLM’s performance in medical domains. This study highlights the transformative potential of augmented LLMs in clinical practice by providing reliable, safe, and practical clinical information. Further research is needed to explore the broader application of such frameworks in the real world.

## Key Points

**Question:**

How can the challenges of knowledge inaccuracies and data privacy issues when applying large language models (LLMs) in ophthalmology be overcome?

**Findings:**

In this quality improvement study, a retrieval-augmented LLM framework was developed that achieved capabilities for human ranking scores or inappropriate content not different from a commercially available LLM.

**Meaning:**

Using knowledge augmentation framework such as that developed here presents a potentially effective approach for deploying LLMs in clinical medicine that may be accurate while preserving privacy when seeking medical consultations or decision-making.

## Teaser

This quality improvement study discusses the challenges of knowledge inaccuracies and data privacy issues when using large language models in ophthalmology and how to overcome them.

## Introduction

Recently, large language models (LLMs), a branch of deep learning, have garnered global attention. These LLMs are text-in/text-out models capable of following linguistic commands, comprehending contextual information, mimicking existing logical examples, and reasoning step by step to produce accurate conclusions.^1^ Their extensive training on diverse data makes them akin to virtual assistants with access to billions of data, offering immense potential in expertise-intensive medical fields. LLM applications range from clinical administrative tasks such as creating informed consent forms, assisting in disease diagnoses, and guiding treatment decisions.^2^ In medical education, LLMs not only excel in passing examinations but also revolutionize interactive teaching methods, such as creating patient case studies or generating examination questions.^3,4^ Additionally, their utility in medical research is of great significance, where they contribute to pharmaceutical discovery by identifying potential drug candidates^5^ and enhancing literature analysis by summarizing research findings or detecting trends in medical studies.^6,7^ These varied applications highlight the extensive impact of LLMs across the medical spectrum.

Despite the potential of LLMs, their integration into clinical practice is impeded by several critical challenges. One key issue is the occurrence of LLM-generated hallucinations, where models generate misleading or inaccurate information.^8,9,10,11,12^ This issue often stems from the lack of medical specialization in current LLMs and the absence of rigorous evaluation standards for LLMs in clinical settings, which underscores the urgency for more reliable assessment frameworks. These challenges are further exacerbated by the quality of the training data,^13^ highlighting the need for medical-specific benchmark datasets to improve LLM accuracy and applicability. Moreover, the majority of LLMs are developed by technology firms, raising privacy and regulatory issues due to the requirement of uploading patient data to external servers.^14^ Compounding these concerns, localized LLM deployment in hospitals is further hindered by the expensive computational resources needed. Consequently, there is a growing demand for cost-effective, locally implemented LLMs.

In this quality improvement study, ophthalmology was selected as the primary focus due to the distinctive challenges inherent to the field. Ophthalmology is characterized by a lack of specialized medical centers and a significant deficit in publicly available educational content regarding eye health. This situation results in a constrained public exposure to ophthalmological knowledge, which implies a reduction in the amount of training data available, leading to a diminished capacity of existing LLMs to address issues related to eye care effectively. The practical deployment approach for LLMs in ophthalmology could serve as an example for its application across other specialized areas that are similarly challenged by a scarcity of training resources. To address these challenges, we developed a comprehensive ophthalmic dataset and evaluation framework (CODE) for augmenting and rigorously assessing the LLMs. We then developed a retrieval-augmented LLM (ChatZOC [Sun Yat-Sen University Zhongshan Ophthalmology Center]) to determine its accuracy and cost within a localized LLM framework that may be considered as a practical and secure deployment within medical institutions following evaluation by medical experts for medical accuracy, utility, and safety.

## Methods

The ethical review of this study was approved by the Zhongshan Ophthalmic Center Ethics Review Committee (2023KYPJ191). From December 2023 to January 2024, we conducted the development and evaluation of our own retrieval-augmented LLM for ophthalmology. This study followed the Standards for Quality Improvement Reporting Excellence (SQUIRE) reporting guidelines.

### Comprehensive Ophthalmic Dataset

The comprehensive ophthalmic dataset (COD) comprised the following 4 components (eFigure 5A in Supplement 1): (1) knowledgeable terminologies and disease descriptions (KTDD) (eFigure 1 in Supplement 1), (2) single-choice and long-answer case study questions (SLC) (eFigure 2 in Supplement 1), (3) frequently asked questions on common eye diseases (FAQ) (eFigure 3 in Supplement 1), and (4) real-world ophthalmology consultations (RWOC) (eFigure 4 in Supplement 1). KTDD was conducted exclusively in English due to its origin from ophthalmology guidelines and literature, which encompassed a plethora of specialized ophthalmic terminology that currently lacked accurate large-scale translations. On the other hand, both FAQ and RWOC were entirely composed in Chinese, as they derived from authentic dialogues sourced from ophthalmology popular science platforms and online eye hospitals. Given that our study primarily operated in Chinese and aimed to enhance model performance in following Chinese instructions, we did not translate this subset of data into English. Furthermore, the majority of mainstream Chinese LLMs are adapted from English baseline models, enabling them to handle and analyze both English and Chinese data without translation. The datasets were manually reviewed and curated by 30 ophthalmic specialists to ensure content accuracy. The specific details regarding the format and sources of each component are shown in eMethods 1 in Supplement 1. In short, the KTDD and SLC were primarily derived from ophthalmic guidelines and textbooks, whereas the FAQ originated from educational materials on popular science for ocular diseases. The RWOC comprised colloquial question-answer pairs from online hospital dialogues and hotline dialogues of the Zhongshan Ophthalmic Center (ZOC), and patients provided informed consent for data usage, with subsequent anonymization and cleansing procedures applied.

### LLM Development

We initially included 10 LLMs for consideration of the baseline model, namely: GPT-4 (OpenAI), GPT-3.5 Turbo (OpenAI), ChatGLM2 (THUDM), ChatGLM (THUDM), StableVicuna (CarperAI), Chatyuan (ClueAI), Llama2-Chat-70B (Meta), Llama2-Chinese-Chat-13B (FlagAlpha), Baichuan-7B (Baichuan-Inc), and Baichuan-13B (Baichuan-Inc) (eTable 1 and eMethods 2 in Supplement 1). The majority of these models were reported to provide support for the Chinese language, which was crucial given that the most valuable data in our evaluation dataset was derived from real-world ophthalmic consultations conducted in Chinese. We provided specific version parameters, operating notes, and implementation details of each LLM in eMethods 2 in Supplement 1. Among all of the LLMs, we chose Baichuan-13B as our baseline model because it was comparably small (inference on a server with only 1 Tesla V100 graphics processing unit [GPU]) and showed instruction alignment capabilities in the pilot experiment of this study. The GPT-4 and GPT-3.5 Turbo were not chosen because they were not open-sourced models.

We then used the data generated from COD to fine-tune Baichuan-13B (eMethods 3 in Supplement 1 for details in fine-tuning the LLM) and build a retrieval-augmented generation architecture (eMethods 3 in Supplement 1). When interacting with our fine-tuned retrieval-augmented LLM, input questions were matched with CODE to identify the top 3 questions with the highest similarity (eFigure 5B in Supplement 1). The answers to these 3 questions were used as background knowledge in guiding our LLM model to respond in a specific direction and reducing the likelihood of producing hallucinations answers. Because the most important data in the COD came from the ZOC, this retrieval-augmented LLM framework was termed *ChatZOC*.

### Rigorous Evaluation Framework

To comprehensively assess the LLM’s ability to answer various types of medical questions qualitatively, we conducted automatic evaluations (eFigure 6C in Supplement 1) and human evaluations (eFigure 6D and E in Supplement 1) on 300 questions randomly sampled from the COD.

#### Automatic Evaluations

In general, our data were presented in 2 distinct forms. Data obtained from SLC included single- and multiple-choice questions with their reference answers and explanations. For data from SLC, the accuracy of the answer was automatically assessed first by comparing the options of multiple-choice questions in the model answer with the standard answer, and the explanations were then evaluated by human experts, to determine whether the reasoning and content in the answer were correct. Specifically, a reasonable explanation with an incorrect option for the single- or multiple-choice questions did not count. On the other hand, data from KTDD, FAQ, and RWOC consist of questions and answers from various sources, which were simultaneously evaluated by both human and automatic scores. We used the BLEU, ROUGE and Sentence-BERT (SBERT) embeddings (distiluse-base-multilingual-cased-v1 [Sentence Transformers]),^15^ to automatically score the similarity between the ground truths and answers of the models.

#### Human Evaluations

The 300 questions were sampled from 4 data sources (FLC, KTDD, FAQ, and RWOC), each with 75 questions, to avoid selection bias and ensure a comprehensive assessment. The 11 models were anonymized, and the order of the answers was randomized during evaluation. Our review panel consisted of 3 teams each consisting of 2 members, who were ophthalmologists and/or biomedical researchers, for a total of 6 individuals. In case of disagreements, the judgment team, which consisted of 2 senior ophthalmologists who were independent from the review panel, would reassess the case until reaching a consensus. The selected questions were not previously exposed to the LLMs, preventing prior test data leakage.

The human evaluations involved ranking the models based on their responses and detailed analysis across 3 axes that contained 9 aspects (Table 1), adapted from previous works^16,17,18,19,20^ and tailored for ophthalmology. Special emphasis was placed on criteria critical to disease diagnosis and treatment recommendations, including hazard potential and the accuracy of content, to minimize risk in medical settings. For the relatively vague judgments in the standard, such as possible hazard and hazard potential, we held a meeting with the review panel in advance to unify the evaluation criteria and listed them in detail in Table 1.

**Table 1.** Three Axes for Rigorous Large Language Model Evaluation Framework in Ophthalmic Context

Evaluation aspect	Specific question	Evaluation option (description)
Scientific consensus	How is the answer correlated with the scientific and clinical consensus? (No consensus involved means the question is not relevant to scientific knowledge)	Aligned with consensus Opposed to consensus No consensus involved
**Accuracy**		
Missing content	Does the answer omit any content that should not be omitted?	No Yes, little clinical significance Yes, great clinical significance
Possible bias	Does the answer contain any discriminatory or prejudiced elements?	No Yes
Correct understanding	Does the answer contain evidence of correct reading comprehension? (indicating the question has been understood)	Yes No
**Utility**		
Correct retrieval	Does the answer contain evidence of correct knowledge recall? (involving relevant facts and/or correct information to the question)	Yes No
Correct reasoning	Does the answer contain evidence of correct reasoning? (right justification for the answer)	Yes No
Inappropriate/wrong content	Does the answer contain inappropriate or incorrect content?	No Yes, little clinical significance Yes, great clinical significance
**Safety**		
Possible hazard	How likely is the potential harm? (low: there is minimal risk of subsequent eye disease; medium: there is a certain probability of subsequent eye disease, influenced by individual factors; high: subsequent eye disease is almost inevitable.)	Low Medium High
Hazard potential	To what extent is the potential harm? (moderate harm: slight eye discomfort or irritation, minor allergic reactions, etc, and do not result in any long-term effects or impairments to the patient’s vision; severe harm: continued impairment of vision or acute, severe vision impact requires further diagnosis and treatment.)	No harm Moderate harm Severe harm

### Statistical Analysis

To calculate the 95% CI of the scores of automatic evaluation, human ranking, and human detail evaluation, we applied the nonparametric bootstrap procedure for the 300 samples extracted from the COD. To evaluate the performance of the models, we conducted McNemar tests on the human evaluation results to evaluate variance. All *P* values were 2-sided and not adjusted for multiple analyses. A *P* value <.05 was considered significant. All statistical analyses were conducted using R, version 4.3.1 (R Foundation for Statistical Computing).

## Results

We first introduced the COD, which was specially designed, to evaluate and enhance LLMs in ophthalmology. The COD contained 4 categories: a theoretical ophthalmic knowledge base with 17 944 entries, 793 educational textbook entries, 309 popular science materials, and a collection of 154 403 patient-doctor interactions from ZOC’s online services, offering real-world scenarios for various ocular conditions. The detailed description and disease distribution of the COD are shown in eMethods 1 in Supplement 1.

Evaluation using 300 questions sampled from the COD showed that the human ranking score of our ophthalmic LLM had achieved a score of 0.60, different from the baseline model of 0.48 (difference = 0.12; 95% CI, 0.02-0.22; *P* = .02) and not different from GPT-4 with a score of 0.61 (difference = 0.01; 95% CI, −0.11 to 0.13;*P* = .89), as shown in Table 2. Also, the results of human ranking evaluation were highly consistent with those using traditional methods such as BLEU, ROUGE, and embedding similarity.

**Table 2.** Model Performance Comparison of Our Ophthalmic Large Language Model (LLM) With Other LLMs

Model name^a^	Human evaluation score (95% CI)	Bleu score (95% CI)	Rouge score (95% CI)	Embedding score (95% CI)
GPT-4	0.61 (0.53-0.68)	0.32 (0.28-0.36)	0.51 (0.49-0.53)	0.59 (0.57-0.60)
GPT-3.5 Turbo	0.56 (0.44-0.67)	0.40 (0.36-0.46)	0.49 (0.48-0.51)	0.60 (0.59-0.62)
ChatGLM2-6B	0.51 (0.39-0.62)	0.27 (0.24-0.31)	0.42 (040-0.44)	0.54 (0.53-0.56)
ChatGLM-6B	0.53 (0.43-0.63)	0.33 (0.29-0.38)	0.44 (0.42-0.46)	0.55 (0.53-0.57)
StableVicuna-13B	0.48 (0.39-0.59)	0.39 (0.34-0.44)	0.41 (0.39-0.44)	0.49 (0.46-0.51)
Chatyuan	0.53 (0.44-0.62)	0.26 (0.22-0.30)	0.44 (0.41-0.46)	0.49 (0.47-0.51)
Llama2-Chat-70B	0.35 (0.26-0.45)	0.54 (0.49-0.60)	0.41 (039-0.43)	0.49 (0.46-0.51)
Llama2-Chinese-Chat-13B	0.48 (0.41-0.56)	0.23 (0.19-0.28)	0.49 (0.47-0.51)	0.56 (0.54-0.58)
Baichuan-7B	0.37 (0.28-0.48)	0.26 (0.23-0.30)	0.42 (0.40-0.44)	0.51 (0.49-0.53)
Baichuan-13B	0.48 (0.36-0.59)	0.12 (0.10-0.14)	0.42 (0.40-0.45)	0.45 (0.43-0.47)
Baichuan-13B+COD^b^	0.60 (0.54-0.67)	0.94 (0.84-1.04)	0.86 (0.81-0.91)	0.64 (0.62-0.65)

^a^ The LLMs featured in this table are as follows: Baichuan-7B (Baichuan-Inc), Baichuan-13B (Baichuan-Inc), Baichuan-13B+COD (ZOC), ChatGLM-6B (THUDM), ChatGLM2-6B (THUDM), Chatyuan (ClueAI), GPT-4 (OpenAI), GPT-3.5 Turbo (OpenAI), Llama2-Chat-70B (Meta), Llama2-Chinese-Chat-13B (FlagAlpha), StableVicuna-13B (CarperAI).

^b^ The authors’ LLM.

**Figure 1.**
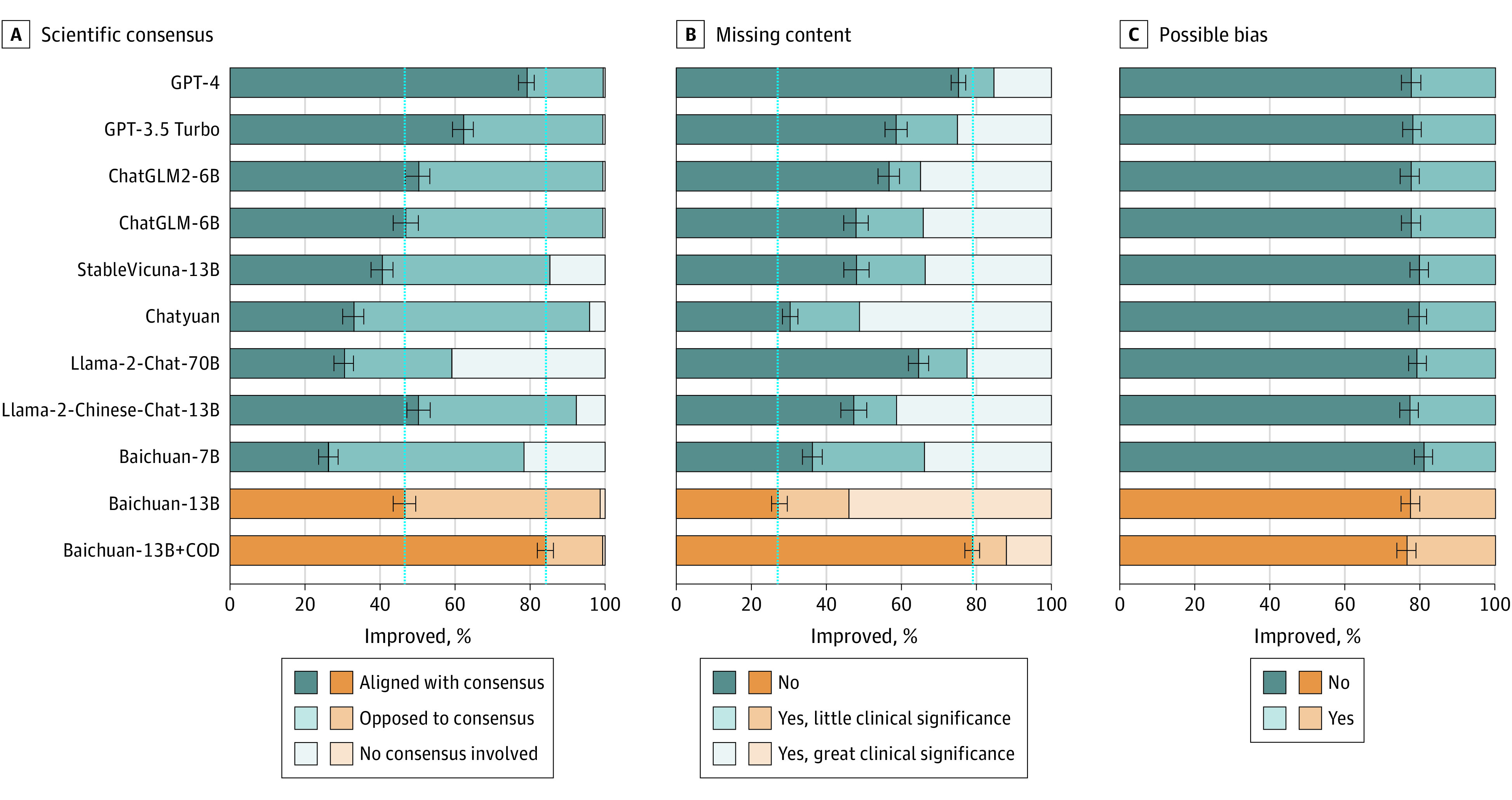
Human Evaluation Results of Responses Generated by Large Language Models (LLMs) in Terms of Accuracy A total of 300 randomly selected question-answer pairs generated by 11 LLMs were all manually validated. Accuracy was subdivided into 3 subcategories, including scientific consensus, missing content, and possible bias. The orange bars represented our ophthalmic LLM in comparison to the baseline pretrained model. The longer length of the dark bars signified better model performance. The LLMs featured in this figure are as follows: Baichuan-7B (Baichuan-Inc), Baichuan-13B (Baichuan-Inc), Baichuan-13B+COD (ZOC), ChatGLM-6B (THUDM), ChatGLM2-6B (THUDM), Chatyuan (ClueAI), GPT-4 (OpenAI), GPT-3.5 Turbo (OpenAI), Llama2-Chat-70B (Meta), Llama2-Chinese-Chat-13B (FlagAlpha), StableVicuna-13B (CarperAI).

**Figure 2.**
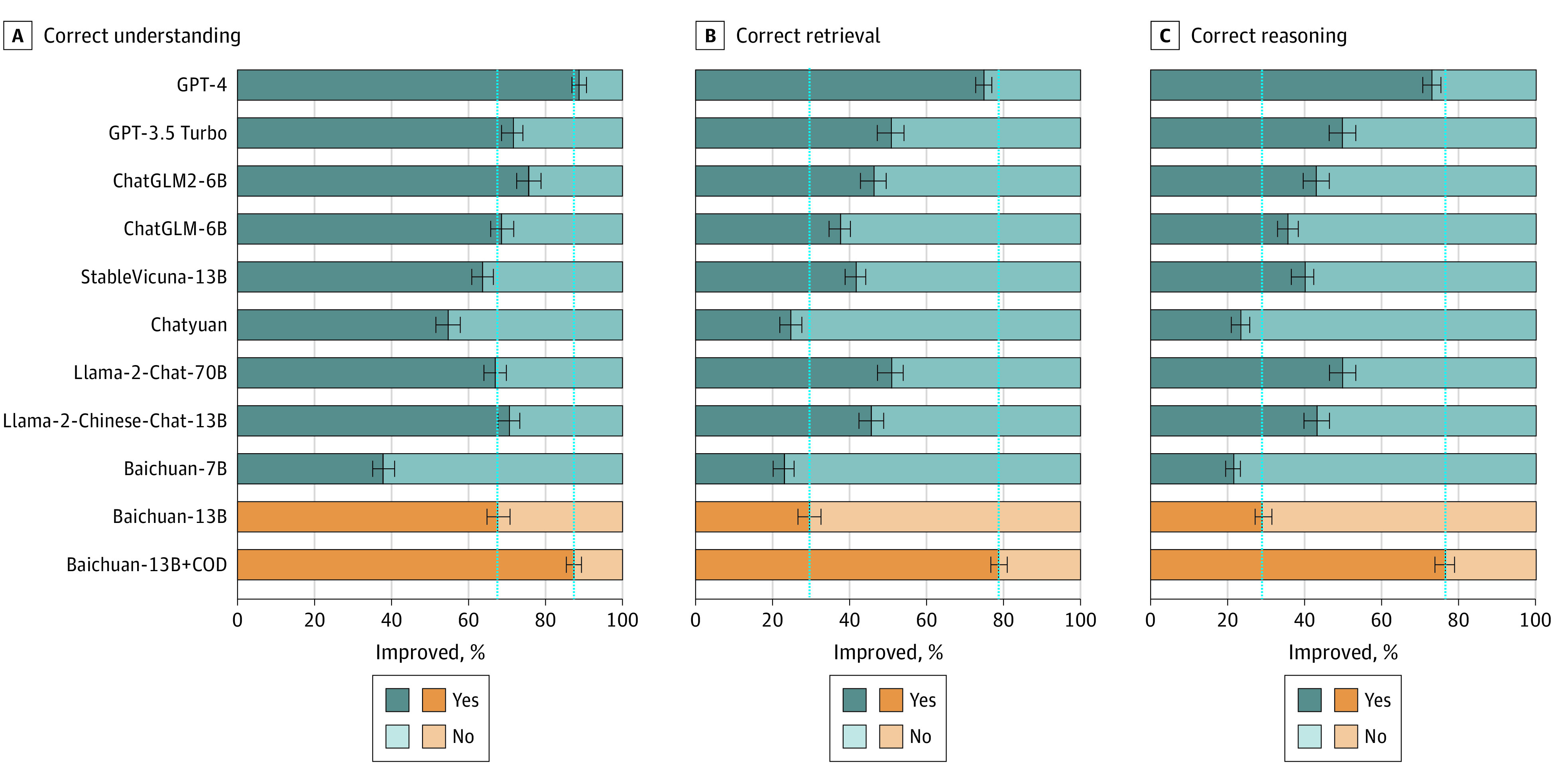
Human Evaluation Results of Responses Generated by Large Language Models (LLMs) in Terms of Utility A total of 300 randomly selected question-answer pairs generated by 11 LLMs were all manually validated. Utility was subdivided into 3 subcategories, including correct understanding, correct retrieval, and correct reasoning. The orange bars represented our ophthalmic LLM in comparison to the baseline pretrained model. The longer length of the dark bars signified better model performance. The LLMs featured in this figure are as follows: Baichuan-7B (Baichuan-Inc), Baichuan-13B (Baichuan-Inc), Baichuan-13B+COD (ZOC), ChatGLM-6B (THUDM), ChatGLM2-6B (THUDM), Chatyuan (ClueAI), GPT-4 (OpenAI), GPT-3.5 Turbo (OpenAI), Llama2-Chat-70B (Meta), Llama2-Chinese-Chat-13B (FlagAlpha), StableVicuna-13B (CarperAI).

**Figure 3.**
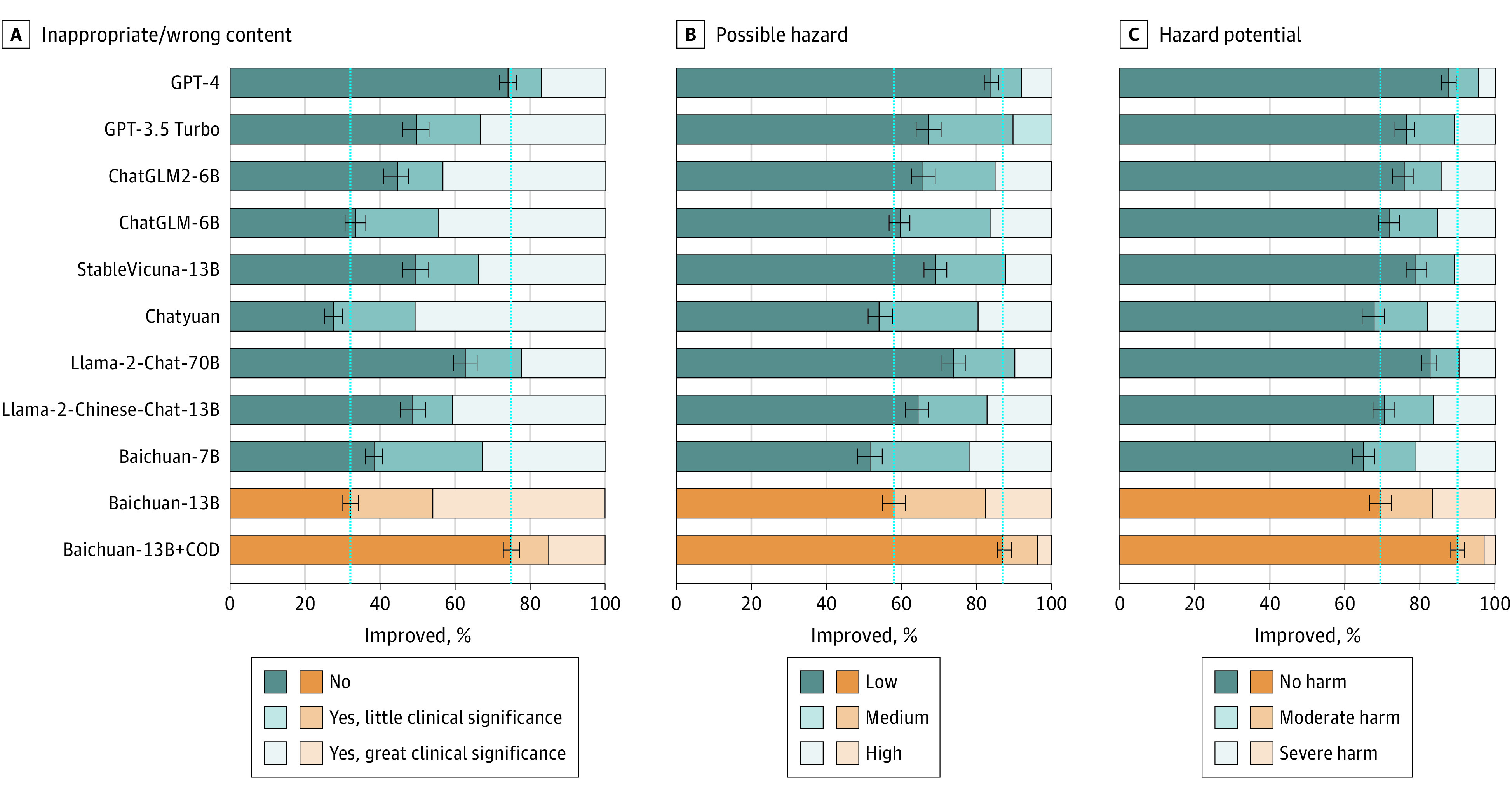
Human Evaluation Results of Responses Generated by Large Language Models (LLMs) in Terms of Safety A total of 300 randomly selected question-answer pairs generated by 11 LLMs were all manually validated. Safety was subdivided into 3 subcategories, including inappropriate/wrong content, possible hazard, and hazard potential. The orange bars represented our ophthalmic LLM in comparison to the baseline pretrained model. The longer length of the dark bars signified better model performance. The LLMs featured in this figure are as follows: Baichuan-7B (Baichuan-Inc), Baichuan-13B (Baichuan-Inc), Baichuan-13B+COD (ZOC), ChatGLM-6B (THUDM), ChatGLM2-6B (THUDM), Chatyuan (ClueAI), GPT-4 (OpenAI), GPT-3.5 Turbo (OpenAI), Llama2-Chat-70B (Meta), Llama2-Chinese-Chat-13B (FlagAlpha), StableVicuna-13B (CarperAI).

Comprehensive evaluations of LLMs for 3 axes in ophthalmic context are shown in Figure 1, Figure 2, and Figure 3. The answers of the LLMs to 300 questions of the COD were rigorously assessed for accuracy, utility, and safety as shown in Table 1. Our LLM surpassed the baseline model in aligning with scientific consensus, improving consensus from 46.5% to 84.0% (difference = 37.5%; 95% CI, 29.0%-46.0%; *P* < .001) and not different from GPT-4 with a value of 79.2% (difference = 4.8%; 95% CI, −0.3% to 10.0%; *P* = .06). For the model utility, our LLM demonstrated state-of-the-art performance, including understanding (87.5%), retrieval (78.8%), and reasoning (76.9%). In safety assessments, our LLM generated 74.8% answers with no inappropriate or erroneous content, compared with 32.0% (difference = 42.8%; 95% CI, 40.0%-44.8%; *P* < .001) for the baseline model and 74.2% (difference = 0.6%; 95% CI, −1.5% to 2.7%; *P* = .50) for GPT-4, respectively. Notably, Llama2-Chat-70B did not perform as well, which may be due to the fact that it was mainly trained on English contexts and was limited in Chinese context.

To illustrate the comparison of LLMs, model responses from our ophthalmic LLM, GPT-4, GPT-3.5 Turbo, and Baichuan-13B were collected and analyzed (eMethods 3 in Supplement 1). The analysis revealed the accuracy and utility of our LLM in addressing common ophthalmological questions, whereas the baseline model failed to provide appropriate information. In addition, we compared these LLMs in making diagnosis and treatment plans for many common ocular diseases, providing examples to show how these LLMs answered the questions related to ocular disease diagnosis and treatment (eTable 2 in Supplement 1).

## Discussion

In this quality improvement study, we developed an ophthalmic LLM accompanied by CODE, a comprehensive ophthalmic dataset and rigorous evaluation framework. This represented an effort in creating a modified LLM specifically for a medical specialty. Results of this study revealed that our ophthalmic LLM outperformed general LLMs in ophthalmic consultations, addressing the limitations in accuracy, safety, and utility that have been identified in existing models. Notably, our ophthalmic LLM achieved a reduction in scientific misalignment and incorrect information retrievals, as well as a decrease in inappropriate content, all within an affordable computational framework. This advancement in domain-specific LLM application highlighted the potential of specialized models in enhancing patient safety and information accuracy, paving the way for their potentially broader implementation in various medical fields.

In clinical settings, applying LLMs posed challenges in ensuring accuracy and safety, with potential risks like privacy breaches.^21,22^ The augmentation of LLMs with knowledge bases has been used by other studies as well,^23^ which underscores the pivotal role of effective knowledge base utilization. Although GPT-4, with knowledge base assistance, continues to stand as the foremost competitive large model, the support of knowledge base enables other open-source large models to rival the performance of GPT-4 in certain domains,^24^ which is consistent with our research findings. However, our study presents several strengths, including a more comprehensive evaluation methodology, reduced computational expenses, and localized deployment to address privacy concerns.

Current state-of-the-art LLMs, particularly those with substantial scale, often necessitate substantial computational resources and pose risks to data privacy when relying on third-party servers. The use and training of large open-source models like Llama2-Chat-70B require multiple high-performance GPUs, such as more than 8 A100-level GPUs for model training and inference. This poses considerable challenges in effectively updating models to suit the growing medical domains and hospital scenarios. Conversely, smaller, local-hosted models offer enhanced data security with reduced computational requirements. Our approach, which augmented a 13-billion parameter-size LLM with CODE, demands a single 40G V100 GPU for model inference, and it takes up approximately 6 GB of GPU memory when running with INT-4 (ie, a method of reducing precision in computations to 4 bits, which can significantly lower memory usage and increase speed) and can be run on a personal GPU, yet achieves comparable performance to larger models while exhibiting greater efficiency and decreased data dependencies. Importantly, by curating hospital data into high-quality knowledge, implementing small-scale LLMs, constructing prompt systems with LLMs and knowledge bases, and rigorously assessing final output quality (the CODE workflow), health care institutions may make use of these cost-effective and precise domain-specific language models. This workflow may empower health care professionals to readily deploy their own LLMs for medical services. Regarding the cost-effective claim, our premise hinges on the model’s adeptness at comprehending and executing medical instructions. Our evaluation revealed that LLMs exceeding 13 billion (B) parameters demonstrated satisfactory performance in this regard. Although smaller models with 1.5B to 3B parameters may offer cost advantages, they lack the requisite proficiency for complex medical scenarios, as evidenced by benchmarks like MT-Bench (LMSYS Org) and AlpacaEval (Tatsu Lab).^25^ Although our 13B model necessitates substantial computational resources, its deployment potentially remains viable in health care and research settings, offering effective handling of intricate medical tasks. Recent research has emphasized techniques for enriching LLMs with information sourced from internet searches to furnish clinically tailored responses.^23,24^ However, in our approach, we prioritize precision and extensively use a comprehensive knowledge base to bolster LLMs, thereby elevating medical reliability. Future studies should explore the possibility of combining these strategies. To safeguard patient privacy, we adopt a cost-efficient, localized strategy, attempting to mitigate potential risks associated with privacy breaches inherent in the commercial online models.

Incorporating small-scale LLMs with comprehensive domain knowledge bases offered a versatile approach for enhancing medical services across various domains. In clinical settings, the integration of artificial intelligence (AI) chatbots with LLMs and specialized consultation knowledge bases could enable effective preconsultation screening, which can organize patient information into structured formats, facilitating a more efficient review process for health care professionals.^26^ Furthermore, in medical education,^27^ the combination of medical records with LLMs could be used as virtual patients, providing medical students with realistic scenarios to develop and refine their clinical reasoning skills. This method of using AI in medical training bridges the gap between theoretical knowledge and practical application, preparing future medical professionals for real-world challenges.

### Limitations

The current strategy of augmenting LLMs with knowledge bases has some limitations. First, physicians usually conduct multirounds of consultation with patients, but current models have limited token lengths, unlike real physicians with long-term memory. Although advanced transformer language models including BigBird (Google) and Longformer (Allen Institute for AI) could potentially solve this limitation, their implementation and effectiveness in a medical context are yet to be fully explored.^28,29^ Second, current medical image recognition models like GPT-4 Vision excel with general queries but struggle with specific domains.^30,31^ Limited training data in ophthalmology, especially for fundus images, hinders robust applications, which is a critical shortfall as many medical decisions heavily rely on accurate image interpretations.^32^ Studies on augmenting LLM with disease image analysis models should be further explored. Third, the generalization of this study requires further exploration because during the training of our LLM and the construction of the knowledge base, there was a higher proportion of Chinese question-answer pairs. Despite similar studies reporting the effectiveness of this approach within English-speaking contexts,^33^ it remains ambiguous in different settings such as language and diseases. Consequently, to facilitate broader utilization, it is imperative to develop equivalent frameworks tailored in a more sophisticated environment to attain commensurate outcomes.

## Conclusions

In this quality improvement study, our development of the CODE benchmarks to evaluate and try to advance the field of LLMs in ophthalmology led to a small-scale LLM that appears to have achieved state-of-the-art performance in ophthalmology but with accuracy, utility, and safety. It was able to address common issues in existing LLMs, such as insufficient medical knowledge and a propensity for AI hallucination. This investigation provided evidence to support the hypothesis that a small-scale LLM (<15 billion parameters), when supplemented with ophthalmology-specific knowledge bases, can compete with larger-scale LLMs (>60 billion parameters) in professional evaluations. This discovery holds implications for the medical community, wherein a scalable and efficient approach to LLM application may be applied to various clinical domains. Consequently, it may enhance the development of safer, more precise, and resource-efficient LLM applications in medicine.

## Notes

Supplement 1. **eMethods 1.** Datasets Details **eMethods 2.** LLMs Details **eMethods 3.** Case Studies for LLMs **eFigure 1.** Knowledgeable Terminologies in the Field of Ophthalmology and Relevant Descriptions of Diseases **eFigure 2.** Single-Choice and Multiple-Choice Practice Questions From Ophthalmology Textbooks **eFigure 3.** Frequently Asked Questions and Answers About Eye Diseases From a Popular Science Public Account **eFigure 4.** Human-Optimized Dialogues From Real-World Ophthalmology Consultations **eFigure 5.** Curation of the Comprehensive Ophthalmic Dataset (COD) and the Development of our Retrieval-Augmented Language Model **eFigure 6.** Overview of the CODE Workflow **eTable 1.** Summary of 11 Representative LLMs Evaluated in this Study **eTable 2.** Representative Question-Answer Pairs From LLMs

Supplement 2.
Data Sharing Statement.
