# Supplementary material for: Development and Evaluation of a Retrieval-Augmented Large Language Model Framework for Ophthalmology
Source: JAMA Ophthalmol. 2024 Jul 18;142(9):798–805. doi: 10.1001/jamaophthalmol.2024.2513 (PMC11258636; doi:10.1001/jamaophthalmol.2024.2513)
Supplement: Supplement 1. — eMethods 1. Datasets Details eMethods 2. LLMs Details eMethods 3. Case Studies for LLMs eFigure 1. Knowledgeable Terminologies in the Field of Ophthalmology and Relevant Descriptions of Diseases eFigure 2. Single-Choice and Multiple-Choice Practice Questions From Ophthalmology Textbooks eFigure 3. Frequently Asked Questions and Answers About Eye Diseases From a Popular Science Public Account eFigure 4. Human-Optimized Dialogues From Real-World Ophthalmology Consultations eFigure 5. Curation of the Comprehensive Ophthalmic Dataset (COD) and the Development of our Retrieval-Augmented Language Model eFigure 6. Overview of the CODE Workflow eTable 1. Summary of 11 Representative LLMs Evaluated in this Study eTable 2. Representative Question-Answer Pairs From LLMs [file jamaophthalmol-e242513-s001.pdf]

Supplementary Online Content

Luo MJ, Pang J, Bi S, et al. Development and evaluation of a retrieval-augmented large language model framework for ophthalmology. *JAMA Ophthalmol*. Published online July 18, 2024.
doi:10.1001/jamaophthalmol.2024.2513

eMethods 1. Datasets Details

eMethods 2. LLMs Details

eMethods 3. Case Studies for LLMs

eFigure 1. Knowledgeable Terminologies in the Field of Ophthalmology and Relevant Descriptions of Diseases

eFigure 2. Single-Choice and Multiple-Choice Practice Questions From Ophthalmology Textbooks

eFigure 3. Frequently Asked Questions and Answers About Eye Diseases From a Popular Science Public Account

eFigure 4. Human-Optimized Dialogues From Real-World Ophthalmology Consultations

eFigure 5. Curation of the Comprehensive Ophthalmic Dataset (COD) and the Development of our Retrieval-Augmented Language Model

eFigure 6. Overview of the CODE Workflow

eTable 1. Summary of 11 Representative LLMs Evaluated in this Study

eTable 2. Representative Question-Answer Pairs From LLMs

This supplementary material has been provided by the authors to give readers additional information about their work.

© Luo MJ et al. *JAMA Ophthalmology*.

eMethods 1: Datasets details

Detailed description of COD, a comprehensive ophthalmic dataset.

Evaluating medical inquiries necessitated advanced reading comprehension, patient information retrieval, and the capability to synthesize medical knowledge and logical reasoning. To appraise the aptitude of language models in ophthalmology, various formatted questions were aggregated as test datasets. Apart from the existing ophthalmological question-answer datasets and textbook case studies for assessing the model's ophthalmological knowledge application, colloquial question-answer pairs centered on popular optometric understanding and realistic clinical asks would also be compiled. The language models would undergo quantitative algorithmic assessments complemented by qualitative manual reviews of their response generation proficiency using these multifaceted eye health queries.

These datasets not only varied in type but also in their sources and languages. We believe that a diverse range of ophthalmological question-answer data from various categories and sources can comprehensively evaluate LLM's abilities in multiple aspects of clinical ophthalmology, including the ability to not only understand and reason with knowledge but also the ability to use precise and metaphorical language for clearer patient communication.

The aggregated datasets exhibited diversity in typology, provenance, and linguistics. Compiling ophthalmological query-response data from multifarious categories and sources is hypothesized to facilitate a comprehensive appraisal of language models across various facets of clinical optometry including comprehension, reasoning, and utilizing precise and figurative vernacular to optimize patient communication. The heterogeneous datasets were postulated to better examine the models' ophthalmological knowledge application through responses tailored to varied settings. The entire dataset consisted of four distinct components (Figure S5), namely: **KTDD** for knowledgeable terminologies in ophthalmology and relevant descriptions of diseases; **SLC** for single-choice and long-answer case study practice questions from ophthalmology textbooks; **FAQ** for frequently asked questions and answers about common eye diseases from a popular science public account; **RWOC** for human-optimized dialogues from real-world ophthalmology consultations in internet hospital. Among these components, KTDD exclusively comprised English data, while SLC included single-choice questions in English and case-based questions in Chinese. FAQ and RWOC both consisted entirely of Chinese data. The COD datasets were reviewed by ophthalmic specialists for content accuracy.

85% of RWOC's content were scenario-related questions about disease diagnosis, treatment plan and prognosis advice for real patients with ocular diseases, following the distribution of 23.4% glaucoma, 22.6% dry eye syndrome, 18.5% conjunctivitis, 16.1% fundus diseases, 12.9% cataract, 4.1% refractive error, and 2.4% other eye conditions.

© Luo MJ et al. *JAMA Ophthalmology*.

eMethods 2: the construction details of our ophthalmic LLM

1. Model evaluated in this work.

GPT-4 (NA, estimate 175B+)

- Version: GPT-4 July 30 Version
- Release: https://chat.openai.com/
- Date: 2023/7/30
- Model type: Close source
- Method: OpenAI API Key calling

GPT-3.5 Turbo (NA, estimate 175B)

- Version: GPT-3.5 Turbo May 24 Version
- Release: https://chat.openai.com/
- Date: 2023/5/24
- Model type: Close source
- Method: OpenAI API Key calling

ChatGLM2 (6B)

- Link:https://huggingface.co/THUDM/chatglm2-6b/tree/b259b27320263629b0afccef134c54028233673d
- Date: 2023/6/27
- Release: b259b27320263629b0afccef134c54028233673d
- Method: Download and run the model on local server. (AMAX XP-48201G with 5*V100 GPU)
- Model type: Open source

Model info: ChatGLM2-6B is the second-generation version of the open-source bilingual dialogue model, ChatGLM-6B. Building upon the excellent features of the original model, such as smooth conversation flow and ease of deployment, ChatGLM2-6B introduces the following new enhancements: 1. Enhanced Performance; 2. Extended Context Length; 3. Improved Efficiency in Inference; 4. More Open Protocols

ChatGLM (6B)

- Link:https://huggingface.co/THUDM/chatglm-6b/tree/6461061e82ba1556f5748ba530e0a33fcbece3ae
- Date: 2023/4/6
- Release: 6461061e82ba1556f5748ba530e0a33fcbece3ae
- Method: Download and run the model on local server. (AMAX XP-48201G with 5*V100 GPU)
- Model type: Open source
- Method: Download and run the model on local server. (4 P40 GPU on TBD)
- Model info: ChatGLM-6B is an open source, Chinese-English Q&A-supported conversational language model based on the General Language Model (GLM) architecture with 6.2 billion parameters. Combined with model quantization techniques, it

is optimized for Chinese Q&A and conversations.

Stable vicuna (13B)

- Link:https://huggingface.co/CarperAI/stable-vicuna-13b-delta/tree/2c8d73c2fe8b42f0fec7855ad1d05658c8e48321
- Release: 2c8d73c2fe8b42f0fec7855ad1d05658c8e48321
- Date: 2023/4/28
- Model type: Open source
- Method: Download and run the model on local server. (AMAX XP-48201G with 5*V100 GPU)
- Model info: StableVicuna-13B is fine-tuned from Vicuna-13B on a mix of three datasets: OpenAssistant Conversations Dataset (OASST1), GPT4All Prompt Generations, and Alpaca. And the reinforcement learning from human feedback (RLHF) was also trained on OpenAssistant Conversations Dataset (OASST1) along with two other datasets: Anthropic HH-RLHF and Stanford Human Preferences Dataset.

Chatyuan (6B)

- Link:https://huggingface.co/ClueAI/ChatYuan-large-v2/tree/313b02664c04bc67afd60c6f11a230faf1a67573
- Date: 2023/4/27
- Release: 313b02664c04bc67afd60c6f11a230faf1a67573
- Method: Download and run the model on local server. (AMAX XP-48201G with 5*V100 GPU)
- Model info: ChatYuan-large-v2 supports both Chinese and English, with optimizations in instruction fine-tuning, human feedback reinforcement learning, and thought chaining with extra conversation data from the LLM model PromptCLUE-large.

Llama2-chat-70B (70B)

- Link (apply): https://ai.meta.com/resources/models-and-libraries/llama-downloads/
- Date: 2023/7/18
- Release: 2023/7/18
- Method: Download and run the model on Supercomputing Center server. (8*A800 GPU)
- Model info: Llama 2, a collection of pretrained and fine-tuned large language models (LLMs) ranging in scale from 7 billion to 70 billion parameters. Our fine-tuned LLMs, called Llama 2-Chat, are optimized for dialogue use cases.

Llama2-chinese-chat-13B (13B)

- Link: https://huggingface.co/hiyouga/Llama-2-Chinese-13B-chat/tree/bf20573c4ef97b2e46bc9e8a7fb0768c78017ee1
- Date: 2023/7/24
- Release: bf20573c4ef97b2e46bc9e8a7fb0768c78017ee1
- Method: Download and run the model on local server. (AMAX XP-48201G with 5*V100 GPU)
- Model info: A bilingual instruction-tuned LoRA model of https://huggingface.co/meta-

llama/Llama-2-13b-hf; Instruction-following datasets used: alpaca, alpaca-zh, open assistant. Training framework: LLaMA-Factory.

Baichuan-7B (7B)

- Link:https://huggingface.co/baichuan-inc/Baichuan-7B/tree/39916f64eb892ccdc1982b0eef845b3b8fd43f6b
- Date: 2023/6/16
- Release: 39916f64eb892ccdc1982b0eef845b3b8fd43f6b
- Method: Download and run the model on local server. (AMAX XP-48201G with 5*V100 GPU)
- Model type: Open source
- Model info: Baichuan-7B is an open-source large-scale pre-trained model developed by Baichuan Intelligence. This model, based on the Transformer architecture, consists of 7 billion parameters and was trained on approximately 1.2 trillion tokens. It supports both Chinese and English languages and has a context window length of 4096. Baichuan-7B has achieved top performance on standard Chinese and English benchmark evaluations (C-EVAL/MMLU) among models of similar size.

Baichuan-13B (13B)

- link:https://huggingface.co/baichuan-inc/Baichuan-13B-Base/tree/72f24a2f2d5925262c29648929a20e53e4c9cebf
- Date: 2023/7/11
- Release: 72f24a2f2d5925262c29648929a20e53e4c9cebf
- Method: Download and run the model on local server. (AMAX XP-48201G with 5*V100 GPU)
- Model info: Baichuan-13B is an open-source, commercially viable large-scale language model developed by Baichuan Intelligence, following the success of Baichuan-7B. With a whopping 13 billion parameters, this model has demonstrated top performance on authoritative Chinese and English benchmarks among models of similar size.

2. The supervised fine-tuning of LLM.

For model supervised fine tuning, our dataset was generated from COD. As most of the data in KTDD is in English, to avoid making the model answer in English, We opted to train on a subset of this data, selecting ocular disease data that is more prevalent based on disease frequency, to constitute our training set. We adopted all FAQ question-answer data, as the FAQ comes from high-quality ophthalmology knowledge. In the RWOC data, we carefully selected question-answer pairs with ophthalmology knowledge and keywords, discarding many data points related to prescribing medications, polite phrases, rhetorical questions, etc., and conducted data deduplication. For the SLC data, we directly concatenated the correct answers into the question’s answer, generating question-answer format data. Finally, we integrated data from four sources, forming a total of 34,503 question-answer pairs in the training dataset.

Regarding the model training process, we utilized 8 Nvidia Tesla A800 GPUs (80GB RAM) and the DeepSpeed framework for Lora-SFT fine-tuning on the Baichuan-13B-Chat

© Luo MJ et al. *JAMA Ophthalmology*.

model with a learning rate of 5e-5 and 4 batches per device. The training lasted approximately 7 hours, with a total of 15 epochs.

3. The construction of Retrieval-Augmented Generation(RAG) architecture.

We utilize the Chroma, an open-source embedding database (https://github.com/chroma-core/chroma), to vectorize the CODE dataset and convert it to a knowledge base. Within the framework of Chroma, we used Sentence Bidirectional Encoder Representations from Transformers (SBERT) embeddings model named distiluse-base-multilingual-cased-v1 (https://huggingface.co/sentence-transformers/distiluse-base-multilingual-cased-v1) as a semantic textual similarity model to match the doctor's query with questions in the database and extract relevant patient information. When interacting with our fine-tuned Retrieval-Augmented LLM, input questions were embedded into vectors and matched with CODE to identify the top three questions with the highest similarity (eFigure S5b). The answers to these three questions were then used as background knowledge in the fine-tuned Baichuan-13B's inquiry prompt, guiding it to respond in a specific direction and reducing the likelihood of producing hallucinations answers.

4. Comparison of computing resources between our RAG LLM and GPT-4

Our approach, augmented a 13 billion parameter size LLM with CODE, fine-tuning by eight 80G A800 GPU for 7 hours on 34503 ophthalmic question-answer pairs, demands a single 40G V100 GPU for model inference, and it takes up about 6GB of GPU memory when running with int-4, and can be run on a personal GPU. While GPT-4 was estimated to have approximately 1.8 trillion parameters across 120 layers, surpasses GPT-3's size by over 10 times. OpenAI incorporates Mixture Of Experts (MoE) in its model, with 16 experts, each possessing around 111 billion parameters for MLP (Multilayer Perceptron). Two of these experts are routed per forward pass, potentially aiding in cost management. Despite its capabilities, GPT-4's inference cost is three times higher than that of the 175 billion parameter Davinci, attributed to the necessity for larger clusters and lower utilization rates. The inference architecture of GPT-4 operates on a cluster of 128 GPUs, employing 8-way tensor parallelism and 16-way pipeline parallelism. (https://the-decoder.com/gpt-4-architecture-datasets-costs-and-more-leaked/)

eMethods 3: Case studies for LLMs

Case 1

Original question (Prompt)

What is Meibomian Gland Massage? What's the process?

Standard answer

Meibomian Gland Massage Method - Hospital Chapter Meibomian gland massage is performed in the hospital. The standard operation is generally as follows: 1. Lie flat on the treatment bed. The doctor uses a sterile cotton swab dipped in saline to clean the roots of your eyelashes and remove the eyelid margins discharge, scab, etc. 2. Apply hot compress. Use a sterile hot towel to apply heat to the eyelids for 5-10 minutes to soften secretions. 3. Massage. The doctor uses special meibomian gland massage forceps (some hospitals will use glass rods, cotton swabs, etc.) to clamp the eyelids, and by constantly pinching the eyelids, squeeze the meibomian glands and squeeze out the secretions; at the same time, use another cotton swab to wipe the secretions. 4. Clean the eyelid margin again. Put some antibiotic eye drops into your eyes or apply eye ointment to your eyelids. Meibomian gland massage is usually performed 1-3 times a week. The doctor will adjust the specific frequency according to the severity of symptoms and the nature of secretions. When you are at home, you can apply hot or cold compresses to your eyes. Hot compresses can soften the meibum, while cold compresses can reduce congestion, making the eyes less red, and can also stimulate tear secretion. So applying it to the eyes can also relieve eye discomfort! Finally, if you experience discomfort such as dry eyes, it is recommended to go to a regular hospital for treatment and perform meibomian gland massage under the guidance and supervision of a doctor! Please do not do it yourself!

In the following LLM-generated answers, medical recommendations that were considered important in the expert review were highlighted.

Model-generated answers

①GPT4

Meibomian gland massage is an ophthalmic treatment method mainly used to treat meibomian gland dysfunction (MGD, Meibomian Gland Dysfunction). Meibomian glands are small oil glands located at the edge of the eyelids that secrete oil to stabilize the fatty layer of the tear film. When these glands become blocked or did not work properly, dry eye symptoms can result. Purpose of Meibomian Gland Massage: To help release and circulate blocked oil. Reduces inflammation within the glands. Improve and enhance the quality of oils and fats. Reduces dry eye and other related symptoms. Meibomian gland massage process: Preparation: First, **you can apply** a warm towel or a special warm eye mask on your closed eyes for about 5-10 minutes. This softens the oil in the gland and makes it easier to drain. Massage method: Gently press the eyelids with clean fingers (usually your index finger). Starting from the corners of the eyes, massage outwards using smooth movements, as if squeezing the glands along the eyelids. Repeat a few times and then do the same with the other eye. When you're done: You can gently cleanse your eyelids with warm water and a

non-irritating eye cleanser or lotion to remove drained oils and possible residual dirt. Note: Keep hands and eyes clean to avoid introducing bacteria. Excessive force or incorrect technique can cause eye injury or worse irritation, so it is recommended to perform or learn massage techniques under the guidance of a professional. If you have inflammation, infection, or other problems with your eyes, or if you feel discomfort after a meibomian gland massage, you should stop immediately and consult an ophthalmologist.

②GPT-3.5 Turbo

Meibomian gland massage is an eye care method designed to promote the patency of the meibomian glands and improve eye health and comfort. Meibomian glands, located on the edges of the upper and lower eyelids, produce oil that helps lubricate the surface of the eyeball and prevent dryness and discomfort. Massaging the meibomian glands can help remove oil blockage, reduce dry eye symptoms, and improve eye health. Here's a common meibomian gland massage routine: Preparation: Wash your hands and make sure they are clean. You can prepare your eyes for massage by moistening them with warm water or applying artificial tears. Use a cotton swab or sterile pad: You can use a clean cotton swab or sterile pad. If your doctor recommends it, you can also apply a doctor-recommended eye lubricant to the cotton swab. Massage the upper eyelids: Gently massage the edge of the upper eyelids with a cotton swab or pad, from the inner corner of the eye to the outer corner of the eye, lasting about 15-20 seconds each time. Massage the lower eyelid: Similarly, gently massage the edge of the lower eyelid, from the inner corner of the eye to the outer corner of the eye, lasting about 15-20 seconds each time. Eye closure: **After the massage is completed, you can close your eyes to allow the secretions of the eyelid glands to be evenly distributed on the surface of the eyeball.** Frequency: As recommended by your doctor, perform meibomian gland massage 1-2 times a day to maintain eye health. Please note that meibomian gland massage should be performed under the guidance of a doctor, especially if you have a history of eye problems or surgery. Improper massage may cause discomfort or other problems. If you are considering a meibomian gland massage, or if you already have eye problems, it is recommended that you consult a professional ophthalmologist for appropriate advice and guidance.

③Baichuan13B (Baseline)

Meibomian gland massage is an ophthalmic treatment method mainly used to treat **styes (also known as styes, hordeolum)** and chalazions. This condition is caused by blockage or infection of the meibomian glands. Massage can help drain blockages from the meibomian glands, reduce inflammation and relieve symptoms.

The following is the basic process for performing meibomian gland massage:

1. Preparation: First, make sure your hands are clean and then put on disposable gloves.
2. Patient preparation: Have the patient sit in a comfortable position with eyes open. If needed, use a lubricant (such as paraffin oil or artificial tears) to help the massage process go more smoothly.
3. Location: Find the location of the meibomian glands. On the inside of the eye, along the groove-like structures at the base of the eyelashes, are the meibomian glands.
4. Massage: Gently pinch your eyelids with your index finger and thumb to find a comfortable

position. Then, use the pad of your index finger to gently press the meibomian glands and massage them in a circular motion. Be careful not to use excessive force to avoid damaging your eyes or skin.
5. Repeat: Repeat the meibomian gland massage according to the patient's condition and response. Usually, it needs to be done 3-5 times, with an interval of about 10 minutes each time.
6. Observe: During the massage, pay close attention to the patient's reactions. If adverse reactions such as increased pain, increased redness and swelling occur, the massage should be stopped immediately.
7. Follow-up treatment: After massage, patients may need antibiotic eye drops or other medications to control infection and inflammation. At the same time, maintaining good living habits and avoiding rubbing your eyes will help speed up recovery.
Please note that while meibomian gland massage is a safe and effective treatment for many people, it is best to seek professional medical advice before proceeding. If you have any concerns, please seek medical advice promptly.

④ChatZOC
Meibomian gland massage is an ophthalmic treatment method, mainly used to treat eye problems caused by meibomian gland dysfunction, such as dry eye syndrome, chalazion, etc. The following is **the general process for meibomian gland massage in the hospital:**
1. The patient lies flat on the treatment bed, and the doctor begins to clean the eyes. He/She uses a sterile cotton swab dipped in saline to clean the roots of the patient's eyelashes and remove eyelid secretions, scabs, etc.
2. After cleaning, the doctor will apply hot compress to the patient, usually lasting 5-10 minutes, to soften the secretions.
3. After applying hot compress, the doctor uses special meibomian gland massage forceps or other tools to clamp the eyelids and squeeze the meibomian glands by constantly pinching the eyelids to help discharge secretions.
4. The doctor will use another cotton swab to wipe the eyelid to remove the squeezed secretions.
5. If necessary, the doctor will also instill a small amount of antibiotic eye drops or apply eye ointment to prevent infection.
6. Meibomian gland massage is usually performed 1-3 times a week. The specific frequency will depend on the severity of the patient's symptoms and the nature of the secretion.
At home, you can also use hot or cold compresses on your eyes to relieve eye discomfort, but please note that if you experience uncomfortable symptoms such as dry eyes, it is best to consult a professional doctor and perform meibomian gland massage under the guidance of a doctor. **Do not do it yourself.**

Case 2

Original question (Prompt)
What are the methods to delay the progression of myopia?

© Luo MJ et al. *JAMA Ophthalmology*.

Standard answer

The first method is medication, such as atropine eye drops. The second is optical means. Glasses are a kind of optical means. There is another kind of corneal plastic lenses (OK lenses), but the use of OK lenses may have potential complications, so be sure to carry out a medical optometry with doctor prescription. The third way is to change your lifestyle, reduce close-work time and increase outdoor time.

Model-generated answers

①GPT-4

Myopia progression, especially during childhood and adolescence, has become a global concern. Here are some suggested ways and strategies to slow myopia progression: Outdoor activity: Studies have shown that 1-2 hours of outdoor activity per day can reduce the risk of myopia in children. Outdoor light may have a moderating effect on eye growth. Reduce sustained near work: Prolonged reading, writing or use of electronic devices may be associated with the development and progression of myopia. It is recommended that after every 20-30 minutes of looking at near work, you look away to relax your eyes for about 5 minutes. **Low-dose atropine eye drops**: Some studies have found that low-dose atropine eye drops can slow the progression of myopia. However, use of this method may require a doctor's recommendation and regular checkups. Orthokeratology and multifocal contact lenses: These specially designed eyeglasses and contact lenses are designed to change the focus of light at the back of the eye, thereby slowing the growth of the eye. Ortho-K (Orthokeratology Keratomileusis): These are specially designed contact lenses that are worn at night to temporarily change the shape of the cornea, allowing you to see clearly during the day without the need to wear eyeglasses or contact lenses. It is also thought to slow the progression of myopia. **Controlling eye growth**: Some newer treatments, such as **minimally invasive surgery,** aim to control eye growth and thus slow the progression of myopia. Proper visual habits: Maintain proper reading posture, keep enough light, avoid reading in moving vehicles, etc. Regular eye exams: Especially for children and adolescents, regular eye exams can detect and correct vision problems in time, thus slowing down the progression of myopia. Nutrition: Although there is no clear evidence that certain foods can prevent myopia, a well-balanced diet and adequate vitamins and minerals are still important for eye health. If you or your child has myopia, it is best to discuss with your eye doctor the appropriate strategies and treatments to slow the progression of myopia.

②GPT-3.5 Turbo

Ways to slow the progression of myopia include the following: Control eye time: Reduce prolonged close-up eye use, especially reading electronic devices or books, to avoid overstressing the eyes. Give your eyes a chance to rest by looking away from your eyes at regular intervals. Maintain correct eye posture: Ensure that you maintain the correct eye posture when using your eyes, and do not lower your head too much or tilt your head to look at objects, to avoid adverse effects on your eyesight. Balance indoor and outdoor light: Make sure indoor light is bright and comfortable, avoid excessive light exposure, and engage in more outdoor activities to allow your eyes to fully adapt to natural light. Enhance outdoor activities: Increase

time spent outdoors, as studies have shown a link between outdoor activities and the onset and progression of myopia. Pay attention to eye distance: When using electronic devices, keep a certain distance, usually more than 50 centimeters away from the screen is appropriate. Eat a balanced diet: Provide foods rich in vitamins A, C and E, zinc and omega-3 fatty acids, which contribute to eye health. Regular eye exams: Annual eye exams are recommended for early detection and correction of vision problems. **Reducing risk factors for myopia development: Controlling genetic factors** and avoiding environmental factors, such as reducing prolonged near eye use, can help reduce the risk of myopia development. It is important to note that while these methods can slow the progression of myopia, they do not completely prevent myopia from occurring. If you are already suffering from myopia, timely wearing of doctor-prescribed eyeglasses or contact lenses is an important means of correcting vision problems. If myopia is high or other visual problems occur, special myopia control lenses, etc. may also be considered. Most importantly, maintaining good eye habits and eye care can have a positive impact on protecting your vision and eye health.

③Baichuan13B (Baseline)

1. Control the time of using eyes: reading, watching TV or using electronic products for a long time will increase the risk of myopia, so it is necessary to arrange reasonably and take a break every 45-60 minutes to give your eyes a full rest. 2. Maintain correct eye posture: When reading and writing, keep the distance from the book or screen at least 30 cm, and avoid lying down to read or use cell phones. 3. Adequate light: When reading and writing, have enough light and avoid using your eyes in an environment with too much or too little light. 4. Regular vision check: Check your vision regularly and take timely measures, **such as wearing glasses or contact lenses, to slow down the progression of myopia once vision loss is detected.** 5. Outdoor activities: Participate in outdoor activities more often to increase the time your eyes are exposed to natural light, which can help prevent myopia. 6. Balanced diet: Maintaining a balanced diet and eating foods rich in vitamins A, C and E, such as carrots, green leafy vegetables and nuts, can help protect your eyes. 7. Sufficient sleep: Ensure sufficient sleep to help eyes recover and reduce fatigue. 8. Eye exercises: Performing some eye exercises, such as eyeball rotation and eye massage, can help relieve eye fatigue. 9. Follow the principle of “20-20-20”: look at a place 20 feet (about 6 meters) away for 20 seconds every 20 minutes to help reduce eye fatigue. 10. Wear appropriate glasses: If you are already nearsighted, wear appropriate glasses to slow down the progression of nearsightedness.

④ChatZOC

1.The use of **low concentrations of atropine eye drops** is an effective way to slow down the progression of myopia. Atropine relaxes the eye muscles and reduces eye fatigue, thus slowing down the progression of myopia. However, **you should consult your doctor before using atropine and use it under the doctor’s supervision.** 2. Optical means: Wearing eyeglasses or contact lenses is another common method to slow down the progression of myopia. Wearing proper glasses can help correct vision and reduce the burden on the eyes. For adolescents with high myopia, orthokeratology lens (OK lens) may be considered. OK lens temporarily improves vision by changing the shape of the cornea, **but it must be used under a doctor’s supervision**. 3. Lifestyle modifications: Lifestyle changes can also help slow the progression

© Luo MJ et al. *JAMA Ophthalmology*.

of myopia. This includes decreasing the amount of time spent in close work and increasing the amount of time spent outdoors. Outdoor activities expose the eyes to more natural light and reduce the amount of time spent in close-up, thus reducing eye fatigue. In addition, maintaining a good work schedule, getting enough sleep, and maintaining a healthy diet will also help keep your eyes healthy. In conclusion, **delaying the progression of myopia requires a combination of methods**, including medication, optical means and lifestyle modification. When using these methods, **you should follow your doctor's advice** and have regular eye checkups to ensure that your eyes are healthy.

Case 3

Original question (Prompt)

(Multiple choice question) The patient is a 72-year-old female. She had recurring stinging pain and foreign body sensation in her eyes for half a year. Examination: The visual acuity of the right eye was 0.2, and the visual acuity of the left eye was 0.1. The upper eyelid skin of both eyes is loose, the upper eyelid margin is rolled in, the eyelashes are inverted toward the eyeball and rub the cornea, tarsal scars can be seen when the upper eyelid is turned over, the center of the cornea is light gray-white opacity, and pannus can be seen at the upper limbus. The lenses of both eyes are cloudy.

Trachoma is the most common cause of entropion. Combined with the formation of eyelid scarring and upper limbal pannus, it can be basically determined to be cicatricial entropion caused by trachoma.

Please answer the following questions and provide explanations based on the above information:

The next reasonable treatment plan for the patient is

A. Surgical treatment: phacoemulsification of cataract

B. Surgical treatment: Correction of upper eyelid skin laxity

C. The patient is old and does not consider surgery. Instead, choose drug treatment and give local antibiotic eye drops combined with artificial tears.

D. Surgical treatment: partial tarsal resection of the upper eyelid

E. Short-term drug treatment can be used to relieve symptoms before surgical treatment.

Standard answer

D,E

Cicatricial entropion must be treated surgically, and the surgical method is partial tarsal resection to relieve the traction of tarsal scars. The patient can undergo cataract phacoemulsification surgery, but the current timing of the surgery is not right. Intraocular surgery can only be performed after the entropion and trichiasis of the eyelids are corrected.

Model-generated answers

①GPT4

According to the above description, the patient's main problem is scarring due to entropion, which causes reverse eyelash growth and irritation of the cornea. Therefore, the main treatment

goals are to repair the entropion and resolve the friction between the cornea and eyelashes. Below is an explanation of the options: A. Surgical Treatment: Phacoemulsification - This is a surgery to treat cataracts, although the patient has cataracts, **the main problem at hand is entropion**. This option does not address the primary cause of eye pain and foreign body sensation. B. Surgical treatment: Upper eyelid skin laxity correction surgery - This surgery can help relieve the problem of upper eyelid entropion caused by skin laxity. C. The patient is old and is not considering surgery, opting for medical treatment, giving topical antibiotic eye drops combined with artificial tears - although medical treatment can temporarily relieve symptoms, it does not solve the problem permanently or completely. D. Surgical treatment: Partial tarsal resection of the upper eyelid - This is usually not the first choice procedure for treating entropion. E. Short-term medication can be used to relieve symptoms before surgical treatment - Before surgery, consider using antibiotic eye drops and artificial tears to relieve symptoms and reduce the risk of infection. In summary, **reasonable treatment options include:**
B. Surgical treatment: Correction of upper eyelid skin laxity
E. Short-term medication can be used to relieve symptoms before surgical treatment. Therefore, the correct answers are B and E.

②GPT-3.5 Turbo
Based on the patient's symptoms and examination results, **the next reasonable treatment plan is: B**. Surgical treatment: Correction of upper eyelid skin laxity, often called upper eyelid revision surgery. This surgery is designed to repair entropion and reduce the friction of eyelashes against the cornea, thereby relieving stinging, foreign body sensation and protecting the cornea from further damage. E. Short-term drug treatment can be used to relieve symptoms before surgical treatment, such as topical antibiotic eye drops combined with artificial tears. This can reduce symptoms before surgery, but surgery remains the mainstay of treatment. **Phacoemulsification (A) is not the primary treatment for the present problem because the primary problem is entropion and corneal problems, not the cataract.** Additionally, patients need to address eyelid problems first before considering the need for cataract surgery. Surgical Treatment: Partial tarsal resection of the upper eyelid (D) is usually not the treatment of choice, as upper eyelid skin laxity correction is usually the preferred approach. The patient's age (C) is not the primary factor in deciding whether to undergo surgical treatment, but rather the patient's ocular condition and symptoms determine treatment options. In this case, surgical treatment is a more appropriate option.

③Baichuan13B (Baseline)
This question tests the ability to analyze and process clinical cases. First, we need to understand the patient's condition and causes, and then conduct corresponding diagnosis and treatment for different diseases. From the title, we can learn that the elderly female patient had symptoms such as repeated tingling and foreign body sensation in both eyes. After a series of examinations, it was found that the cause was cicatricial entropion caused by trachoma. Therefore, we should take appropriate treatments to solve this problem. Among the options, there are several possible treatments:
A. Phacoemulsification: This is a method used to treat cataracts and **has nothing to do with this case**.

B. Upper eyelid skin laxity correction surgery: This surgery is mainly used to correct the problem of upper eyelid skin laxity, but it cannot solve the problem of entropion.
C. Medical treatment: Although the patient is old, drug treatment such as topical antibiotic eye drops combined with artificial tears may achieve a certain effect.
D. Partial tarsal resection of the upper eyelid: This is one of the effective methods to solve the problem of entropion.
E. **Short-term use** of medication to relieve symptoms before surgical treatment: **This option is included in option C** because it describes a treatment process rather than a final treatment plan.
Based on the above analysis, **the answer should be D** (surgical treatment: partial tarsal resection of the upper eyelid).

④ChatZOC
The patient's treatment plan should be surgical treatment: partial tarsal resection of upper eyelid. Although the patient is old, cicatricial entropion must be treated surgically **to relieve the traction of the tarsal scar.** The patient can undergo cataract phacoemulsification surgery, but **the current timing of the surgery is not right**. Intraocular surgery can only be performed after the entropion and trichiasis of the eyelids are corrected. Therefore, **options D and E are correct.**

Supplementary Figures

1. eFigure 1

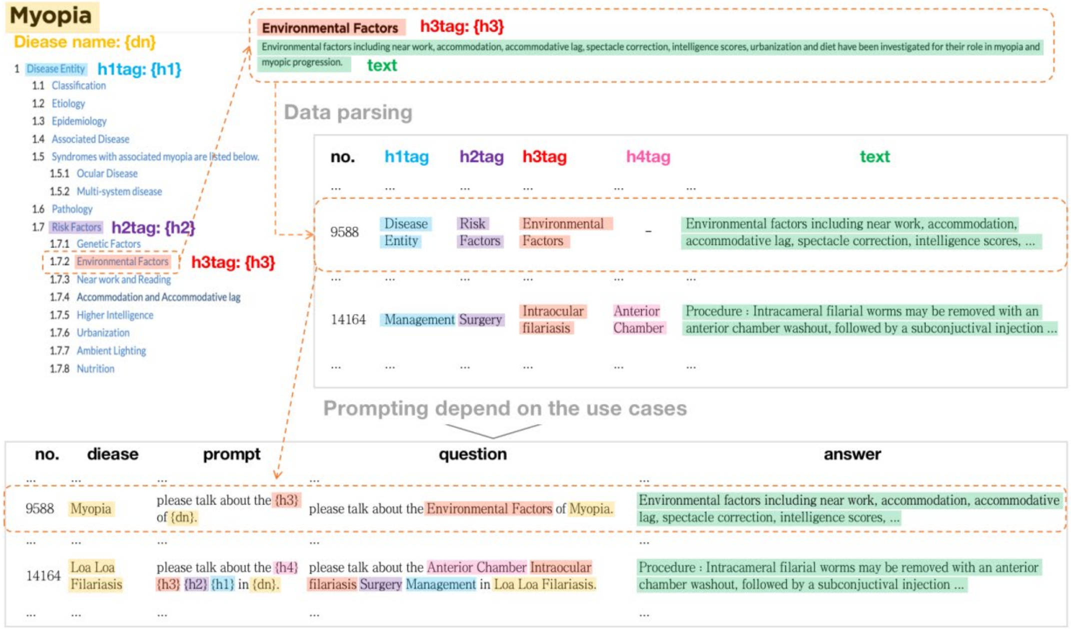

eFigure 1: Knowledgeable terminologies in the field of ophthalmology and relevant descriptions of diseases. This database, intended for academic research purposes only, sourced from Eyewiki, comprises a total of 17,944 prompts generated QAs based on the hierarchical structure of various article titles. Prompts with similar title structures are reused.

© Luo MJ et al. *JAMA Ophthalmology*.

This database is mainly used to assess whether current large language models possess sufficiently detailed knowledge of ophthalmology.

2. eFigure 2

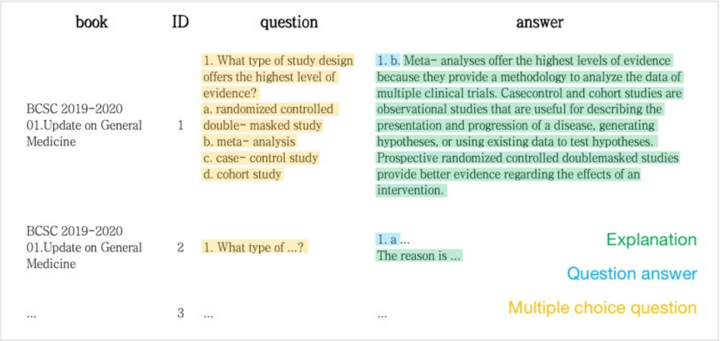

eFigure 2: Single-choice and multiple-choice practice questions from ophthalmology textbooks. This database consists of post-exercise questions from ophthalmology textbooks, totaling 500 multiple-choice questions and 293 multiple-choice case study questions, intended for academic research purposes only. We use “Please pick an answer to the following question first and explain why you choose it” as the prompt. The multiple-choice questions are highlighted in yellow, the answers are highlighted in blue, and the answer explanations are highlighted in green. This database is used to assess whether current models possess knowledge of ophthalmology and the ability to answer questions based on case studies.

3. eFigure 3

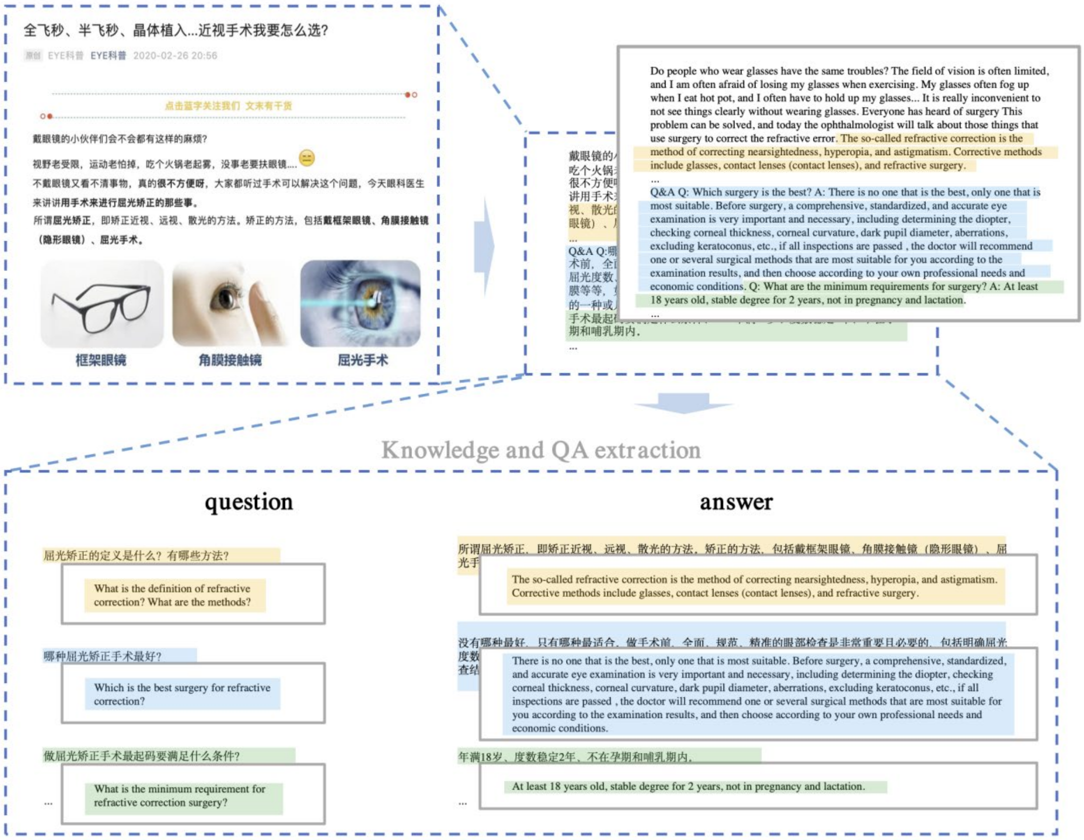

eFigure 3: Frequently asked questions and answers about eye diseases from a popular science public account. This database is derived from 152 popular science articles in ophthalmology, comprising a total of 309 Q&A entries. It was authored by ophthalmologists from Zhongshan Ophthalmic Center, and were meticulously crafted by experienced clinicians at ZOC, using a relatively colloquial and easily understandable language style. The content of the popular science articles includes informative text descriptions about ophthalmology, common Q&A content related to ophthalmology, and ophthalmology-related text within images. The common Q&A content (in blue and green) was directly used to create the Q&A dataset. The informative text descriptions (in yellow) were summarized into Q&A entries using LLMs. Text found within images was manually described to generate Q&A data. This database is intended to assess the LLM model's ability to respond to common ophthalmological questions and colloquial inquiries.

4. eFigure 4

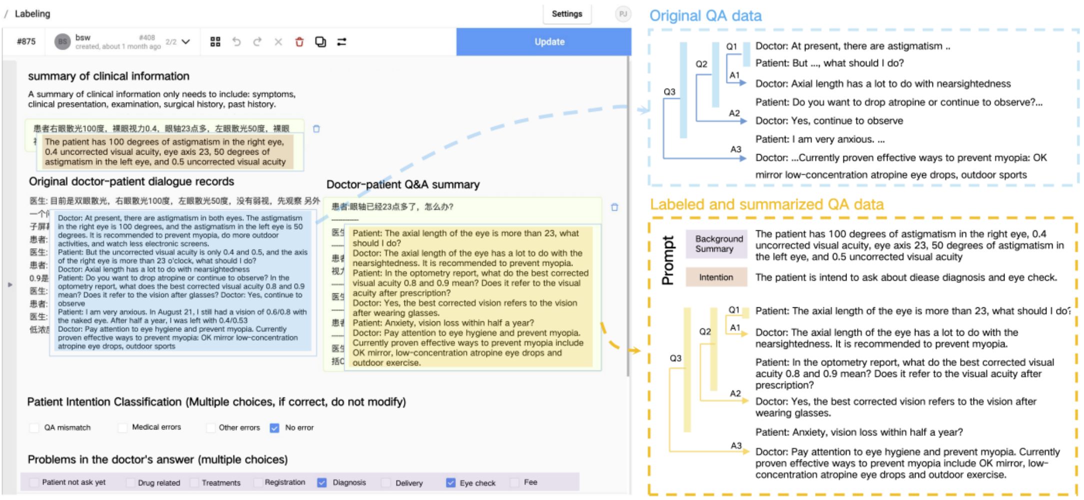

eFigure 4: Human-optimized dialogues from real-world ophthalmology consultations. The database comes from the real dialogue data of Zhongshan Ophthalmic Center's Internet hospitals in 2020-2023, with a total of 100,000+ dialogues, and patients have provided informed consent for data usage, with subsequent anonymization and cleansing procedures applied. Due to the colloquial expression of the real-world dialogue data and the omission of content according to the context, in addition to directly using the original dialogue data to evaluate the real-world dialogue ability of the model, we use Baichaun-13B to summarize the real-world dialogue content and manually correct content and intent annotation to evaluate the model's ability to standardize dialogue.

5. eFigure 5

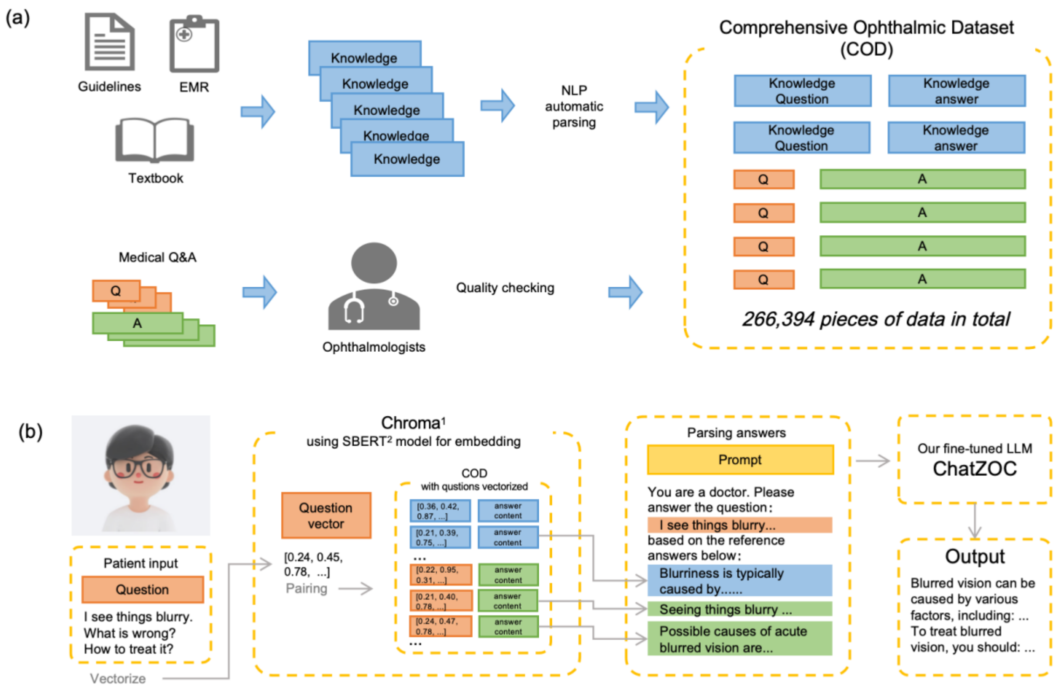

eFigure 5: Curation of the Comprehensive Ophthalmic Dataset (COD) and the development of Our retrieval-augmented language model. (a) The COD encompassed a

diverse array of medical resources, ranging from guidelines and EMR to textbooks, board-style questions, and real-world doctor-patient dialogues. Utilizing in-house NLP methods, this vast information is parsed into question-answer pairs, forming the backbone of the COD's development. (b) Our retrieval-augmented language model combined a pretrained large language model (LLM) with COD knowledge base. Input questions are vectorized and matched with the questions vector in the COD to identify the top three possible answers for the question. This background knowledge guides our fine-tuned LLM to respond in a specific direction, thereby reducing the likelihood of generating responses with AI hallucinations and other potential biases. COD: comprehensive ophthalmic datasets, EMR: electronic medical records, NLP: natural language processing, Q&A: question and answer.
[1]Chroma: The open-source embedding database (https://www.trychroma.com/); [2]SBERT: distiluse-base-multilingual-cased-v1 (https://huggingface.co/sentence-transformers/distiluse-base-multilingual-cased-v1)

6. eFigure 6

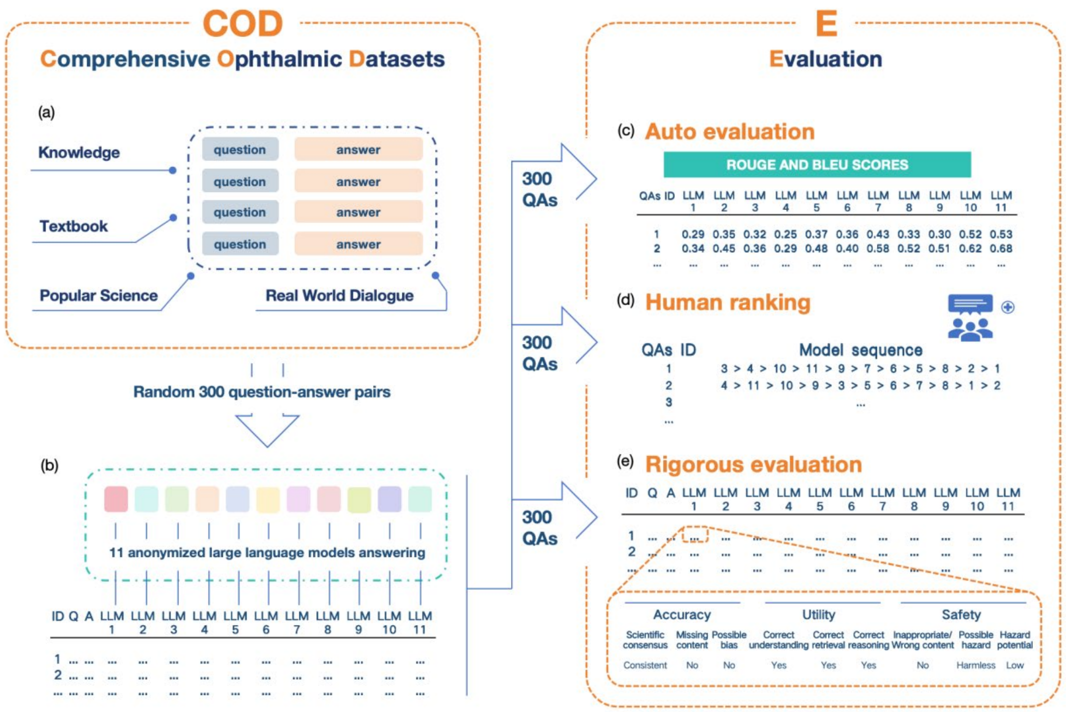

eFigure 6: Overview of the CODE workflow. (a) Data composition of COD (Comprehensive Ophthalmic Datasets). (b) After the dataset transformation, the data was structured into question-answer pairs to the COD. 300 pairs were then submitted to our large language model (LLM) and 10 carefully selected LLMs for response generation. In the Evaluation (E) section, we employed three types of evaluations: (c) an automated evaluation method utilizing ROUGE, BLEU and embedding similarity scores, (d) human ranking evaluation to assesses the overall quality of answers, and (e) comprehensive human evaluation, conducted across three distinct axes, for the 300 random questions and the answers generated by 11 LLMs.

Supplementary tables

eTable 1: Summary of 11 representative LLMs evaluated in this study.

	Model name	Model parameters (Billion)	Computational power cost (inference with NVIDIA Tesla series)	Opensource /Offline usage	Organization & Country
1	GPT-4	NA, estimate Over 175[1]	Over 8 x A100	NO	OpenAI, USA
2	GPT-3.5 Turbo	NA, estimate 175[1]	8 x A100	NO	OpenAI, USA
3	ChatGLM2	6	1 x P40	YES	THUDM, China
4	ChatGLM	6	1 x P40	YES	THUDM, China
5	Stable Vicuna	13	1 x V100	YES	CarperAI, USA
6	Chatyuan	6	1 x P40	YES	ClueAI, China
7	Llama2-chat-70B	70	2 x A800	YES	Meta, USA
8	Llama2-chinese-chat-13B	13	1 x V100	YES	Meta, USA
9	Baichuan-7B	7	1 x P40	YES	Baichuan-inc, China
10	Baichuan-13B (baseline)	13	1 x V100	YES	Baichuan-inc, China
11	ChatZOC (Baichuan-13B + CODE)	13	1 x V100	YES	ZOC, China

1 The model parameters of GPT-4 and GPT-3.5 Turbo were not officially reported. These parameter size were reported by the research community (www.en.wikipedia.org/wiki/GPT-3).

© Luo MJ et al. *JAMA Ophthalmology*.

eTable 2: Representative question-answer pairs from LLMs.

Disease	question	standard answer	GPT-4	GPT-3.5 Turbo	Baichuan-13B (Baseline model)	ChatZOC (LLM+COD)
Cataract	Patient: Male, 65 years old. Underwent right eye cataract phacoemulsification with artificial intraocular lens implantation for cataract. On the first day after the surgery, the right eye had poor vision recovery. Ophthalmic examination: Right eye vision: 0.05, no improvement with correction; Left eye vision: 0.5, no improvement with correction. Mild conjunctival congestion in the right eye, temporal corneal edema, and a membranous object floating in the anterior chamber.<br>Question: Based on the above information, the most likely diagnosis for this patient is:<br>A. Fuchs endothelial corneal dystrophy<br>B. Posterior elastic layer detachment<br>C. ICE syndrome<br>D. Anterior segment toxic reaction syndrome<br>E. Endophthalmitis	B Corneal edema in the early postoperative period after cataract surgery, with a membranous substance seen floating in the anterior chamber suggestive of posterior corneal elastic lamina detachment.	In this 65-year-old patient who presented on the first postoperative day with poor recovery of vision in the right eye, temporal corneal edema, and a membranous float visible in the anterior chamber, we can analyze each of the options:A. Fuchs corneal endothelial dystrophy: while this disease does cause corneal edema, it is a chronic condition that is inconsistent with the rapid onset of postoperative symptoms.B. Posterior elastotic lamina dystrophy: This is a complication that can occur after cataract surgery and usually presents with corneal edema and reduced visual acuity. The membranous material may also be due to damage to the iris or other intraocular tissues as a result of surgical manipulation.C. ICE syndrome: It is a rare syndrome associated with corneal endothelial abnormalities that does not usually occur immediately after cataract surgery.D. Anterior foreground ganglion toxic reaction syndrome: This is an inflammatory reaction in the anterior foreground ganglion due to intraocular	Based on the information provided, the most likely diagnosis for this patient is C. ICE Syndrome (Iridocorneal Endothelial Syndrome).ICE Syndrome is a rare ocular disorder that typically presents with abnormal proliferation and migration of corneal endothelial cells, resulting in blockage of the anterior chamber angle, corneal edema, and vision loss. In ICE syndrome, a membranous substance usually appears to float in the anterior chamber. This syndrome usually affects one eye, such as the patient's right eye, while the left eye is usually unaffected.Treatment of ICE syndrome usually requires corneal transplantation or other surgical interventions to ameliorate corneal edema and vision problems.	Based on the patient's signs and symptoms, the most likely diagnosis is D. Hypersensitivity Reactions of the Anterior Segment (HRA). The patient presents with decreased vision in the right eye, corneal edema, and floating membranous material in the anterior chamber after surgery, all of which are consistent with the manifestations of Hypersensitivity Reactions of the Anterior Segment. Although other options could be the cause, this diagnosis is the most likely based on the information provided.	The most likely diagnosis for this patient is B. Posterior elastic lamina detachment. Based on the information provided, on the first day after ultrasonoemulsification cataract extraction in the right eye, the patient had poor recovery of vision in the right eye, mild conjunctival congestion in the right eye, temporal corneal edema, and a membranous object visible floating in the anterior chamber. These signs and symptoms suggested that the patient might have posterior elastic lamina detachment. Posterior elastic lamina detachment (PELD) is a condition involving the detachment of the posterior elastic lamina in the eye. Treatment options include medication, laser therapy, and surgery. Therefore, based on the information provided, the patient's most likely diagnosis is posterior elastic lamina detachment.

© Luo MJ et al. *JAMA Ophthalmology*.

			medication, such as emulsions used in the surgery, but the description does not quite fit the anterior foreground ganglion E. endophthalmitis: although endophthalmitis may cause vision loss, the description does not mention other typical symptoms of endophthalmitis such as eye pain, headache, iris congestion, or the presence of leukocytes. In summary, the most likely diagnosis is B. Posterior elastic lamina detachment. This is because posterior elastic lamina detachment is a possible complication after cataract surgery, and the patient's symptoms, particularly corneal edema and the presence of membranous material, are consistent with this diagnosis.			
**Disease**	**question**	**standard answer**	**GPT-4**	**GPT-3.5 Turbo**	**Baichuan-13B (Baseline model)**	**ChatZOC (LLM+COD)**
Corneal disease	"Patient: Middle-aged male, experienced a foreign object entering the right eye while sawing a wooden board with a saw 20 days ago, with decreased vision for 2 days. Examination: Vision Vod hand movement/front of the eye, Vos 1.0, NCT: od 8mmHg, os 14mmHg. Mixed conjunctival congestion in the right eye, full-thickness linear penetration in the corneal pupil area about 2mm, corneal edema, mutton-fat-	E Infectious endophthalmitis, if not effectively controlled, the spread of infection leads to total uveitis, which further develops into orbital cellulitis; if the infection spreads to the cavernous sinus via the superior ophthalmic vein causing cavernous sinus embolism, which can be life-threatening, so the most serious complication is cavernous sinus embolism.	Improperly managed, infectious endophthalmitis can lead to a variety of complications. Based on the above description, consider the severity of the following complications:A. Atrophy of the eye: If septic endophthalmitis is not treated promptly or appropriately, the infection can spread further, leading to destruction and atrophy of the eye tissue. This is an extremely serious complication that can lead to complete visual loss.B.	The most serious complication in this situation, if not managed properly, may be:D. Orbital CellulitisAs the patient develops infectious endophthalmitis, if the infection is not effectively controlled, the infection may continue to spread to the tissues surrounding the eye, resulting in orbital cellulitis. Orbital cellulitis is an orbital infection that may lead to severe inflammation and abscess formation around the eye.	E. Cavernous Sinus Embolism EXPLANATION: Based on the patient's description, although there is no detailed information about the cavernous sinus, it is considered that the patient has a serious pathology such as penetrating eye injury, intraocular foreign body, traumatic cataract, and traumatic endophthalmitis, and the possibility of infectious endophthalmitis has developed. Inadequate or untimely	The most serious complication if not managed properly is (E) cavernous sinus embolization. The reasonable management of this patient is (B) vitrectomy + lavage combined with foreign body removal.

© Luo MJ et al. *JAMA Ophthalmology*.

	like KP, a small amount of viscous pus in the anterior chamber, gelatinous exudation in the pupil area, focal posterior synechiae of the iris, completely cloudy lens, and fundus not visible.<br>Considering the history of ocular trauma, clinical signs, and auxiliary examinations, the diagnosis is: right eye perforating injury, intraocular foreign body in the right eye, traumatic cataract in the right eye, and traumatic endophthalmitis in the right eye. The possibility of infectious endophthalmitis in the right eye is high, while sympathetic ophthalmia usually occurs 4-8 weeks after trauma, so it is not considered for diagnosis.<br>Given the symptoms and signs, bacterial infection combined with fungal infection is likely.<br>Vitrectomy combined with foreign body removal and irrigation is indicated.<br>Systemic and local administration of adequate antibiotics combined with antifungal treatment is necessary, avoiding corticosteroids.<br>Question: If not treated properly, the most severe complication could be:<br>A. Phthisis bulbi<br>B. Retinal detachment<br>C. Secondary glaucoma<br>D. Orbital cellulitis<br>E. Cavernous sinus		Retinal detachment: although infection and inflammation can lead to retinal detachment, this is not the most serious outcome when compared to other, more serious complications.C. Secondary glaucoma: infection and inflammation can lead to increased intraocular pressure, but this is not the most serious complication when compared to atrophy of the eyeball.D. Orbital cellulitis: the infection spreading from the the eye to the orbit can lead to orbital cellulitis. This is a serious complication that can lead to visual loss and other complications.E. Cavernous Sinus Embolism: Infection spreading from the eye to the veins of the orbit and then to the cavernous sinus can lead to cavernous sinus embolism. This is an extremely serious complication that can not only lead to loss of vision, but can also be life-threatening as the infection may spread further to the brain. Based on the above analysis, the most serious complication is: E. Cavernous sinus embolism: as this not only involves vision but can be life-threatening for the patient. EXPLANATION: When intraocular inflammation and infection spreads to the orbital veins	This is a serious complication that requires urgent treatment. Complications in the other options are also possible, but orbital cellulitis is one of the most serious and urgent conditions that require urgent treatment, as it can threaten the patient's eye and general health. Therefore, it is crucial to take early and appropriate treatment measures in such cases.	treatment may cause the inflammation to spread into the skull, triggering cavernous sinus embolism, a very dangerous complication that can lead to serious brain infections and other life-threatening conditions. Therefore, option E is appropriate as the most serious complication.	

© Luo MJ et al. *JAMA Ophthalmology*.

	thrombosis"		and further spreads to the cavernous sinus, it can lead to cavernous sinus embolism, which is an acute medical event that requires immediate treatment. Untreated or improperly treated, it can lead to serious complications, such as brain infections, and may even be fatal.			
**Disease**	**question**	**standard answer**	**GPT-4**	**GPT-3.5 Turbo**	**Baichuan-13B (Baseline model)**	**ChatZOC (LLM+COD)**
Myopia	Methods to delay the progression of myopia?	The first is medication, such as atropine, the second is optical means, glasses are also a kind of optical means, there is a kind of corneal plastic lenses (OK lenses), but the use of OK lenses may have potential complications, so be sure to carry out a medical optometry, the doctor recommended to wear before you can wear. The third way is to change your lifestyle, reduce close work and increase time spent outdoors.	Myopia progression, especially during childhood and adolescence, has become a global concern. Here are some suggested ways and strategies to slow myopia progression: Outdoor activity: Studies have shown that 1-2 hours of outdoor activity per day can reduce the risk of myopia in children. Outdoor light may have a moderating effect on eye growth. Reduce sustained near work: Prolonged reading, writing or use of electronic devices may be associated with the development and progression of myopia. It is recommended that after every 20-30 minutes of looking at near work, you look away to relax your eyes for about 5 minutes. Low-dose atropine eye drops: Some studies have found that low-dose atropine eye drops can slow the progression of myopia. However, use of this method may require a doctor's recommendation	Ways to slow the progression of myopia include the following: Control eye time: Reduce prolonged close-up eye use, especially reading electronic devices or books, to avoid overstressing the eyes. Give your eyes a chance to rest by looking away from your eyes at regular intervals. Maintain correct eye posture: Ensure that you maintain the correct eye posture when using your eyes, and do not lower your head too much or tilt your head to look at objects, so as to avoid adverse effects on your eyesight. Balance indoor and outdoor light: Make sure indoor light is bright and comfortable, avoid excessive light exposure, and engage in more outdoor activities to allow your eyes to fully adapt to natural light. Enhance outdoor activities: Increase time spent outdoors, as studies have shown a link between outdoor activities	1. Control eye time: reading, watching TV or using electronic products for a long time will increase the risk of myopia, so it is necessary to arrange eye time reasonably and take a break every 45-60 minutes to give your eyes a full rest. 2. Maintain correct eye posture: When reading and writing, keep the distance from the book or screen at least 30 cm, and avoid lying down to read or use cell phones. 3. Adequate light: When reading and writing, have enough light and avoid using your eyes in an environment with too much or too little light. 4. Regular vision check: Check your vision regularly and take timely measures, such as wearing glasses or contact lenses, to slow down the progression of myopia once vision loss is detected. 5. Outdoor activities: Participate in outdoor activities more often to increase the time	The main methods to slow down the progression of myopia include the following Medication: The use of low concentrations of atropine eye drops is an effective way to slow down the progression of myopia. Atropine relaxes the eye muscles and reduces eye fatigue, thus slowing down the progression of myopia. However, you should consult your doctor before using atropine and use it under the doctor's supervision. 2. Optical means: Wearing proper glasses can help correct vision and reduce the burden on the eyes. For adolescents with high myopia, corneal reshaping lenses (OK lenses) may be considered. Keratoplasty temporarily improves vision by changing the shape of the cornea, but it must be used under a doctor's supervision. 3. Lifestyle modifications: Lifestyle changes can also help slow the progression of myopia. This includes decreasing

© Luo MJ et al. *JAMA Ophthalmology*.

			and regular checkups. Orthokeratology and multifocal contact lenses: These specially designed eyeglasses and contact lenses are designed to change the focus of light at the back of the eye, thereby slowing the growth of the eye.Ortho-K (Orthokeratology Keratomileusis): These are specially designed contact lenses that are worn at night to temporarily change the shape of the cornea, allowing you to see clearly during the day without the need to wear eyeglasses or contact lenses. It is also thought to slow the progression of myopia. Controlling eye growth: Some newer treatments, such as minimally invasive surgery, aim to control eye growth and thus slow the progression of myopia. Proper visual habits: Maintain proper reading posture, keep enough light, avoid reading in moving vehicles, etc. Regular eye exams: Especially for children and adolescents, regular eye exams can detect and correct vision problems in time, thus slowing down the progression of myopia. Nutrition: Although there is no clear evidence that certain foods can prevent myopia, a well-balanced diet and adequate vitamins and minerals are still important for eye health. If	and the onset and progression of myopia. Pay attention to eye distance: When using electronic devices, keep a certain distance, usually more than 50 centimeters away from the screen is appropriate. Eat a balanced diet: Provide foods rich in vitamins A, C and E, zinc and omega-3 fatty acids, which contribute to eye health. Regular eye exams: Annual eye exams are recommended for early detection and correction of vision problems. Reducing risk factors for myopia development: Controlling genetic factors and avoiding environmental factors, such as reducing prolonged near eye use, can help reduce the risk of myopia development. It is important to note that while these methods can slow the progression of myopia, they do not completely prevent myopia from occurring. If you are already suffering from myopia, timely wearing of doctor-prescribed eyeglasses or contact lenses is an important means of correcting vision problems. If myopia is high or other visual problems occur, special myopia control lenses, etc. may also be considered. Most importantly, maintaining good eye habits and eye care can have a positive impact on protecting your	your eyes are exposed to natural light, which can help prevent myopia. 6. Balanced diet: Maintaining a balanced diet and eating foods rich in vitamins A, C and E, such as carrots, green leafy vegetables and nuts, can help protect your eyes. 7. Sufficient sleep: Ensure sufficient sleep to help eyes recover and reduce fatigue. 8. Eye exercises: Performing some eye exercises, such as eyeball rotation and eye massage, can help relieve eye fatigue. 9. Follow the principle of "20-20-20": look at a place 20 feet (about 6 meters) away for 20 seconds every 20 minutes to help reduce eye fatigue. 10. Wear appropriate glasses: If you are already nearsighted, wear appropriate glasses to slow down the progression of nearsightedness.	the amount of time spent in close work and increasing the amount of time spent outdoors. Outdoor activities expose the eyes to more natural light and reduce the amount of time spent in close-up, thus reducing eye fatigue. In addition, maintaining a good work schedule, getting enough sleep, and maintaining a healthy diet will also help keep your eyes healthy. In conclusion, delaying the progression of myopia requires a combination of methods, including medication, optical means and lifestyle modification. When using these methods, you should follow your doctor's advice and have regular eye checkups to ensure that your eyes are healthy.

© Luo MJ et al. *JAMA Ophthalmology*.

			you or your child has myopia, it is best to discuss with your eye doctor the appropriate strategies and treatments to slow the progression of myopia.	vision and eye health.		
**Disease**	**question**	**standard answer**	**GPT-4**	**GPT-3.5 Turbo**	**Baichuan-13B (Baseline model)**	**ChatZOC (LLM+COD)**
Glaucoma	28. What drug can be used to confirm the presence of a Horner pupil?<br>a. Hydroxyamphetamine<br>b. Brimonidine<br>c. Apraclonidine<br>d. Pilocarpine	c. Instilling apraclonidine in both eyes of a patient with Horner syndrome causes reversal of anisocoria due to the development of denervation supersensitivity of the α 1-receptor, which makes the pupil dilator responsive to the weak α1 effect of apraclonidine. Hydroxy-amphetamine is used to localize the Horner syndrome to either pre- or postganglionic. Brimonidine, which is a relatively pure α 2-agonist, has no significant α1-adrenergic effect and therefore does not produce pupil dilation.	Horner's syndrome is characterized by a combination of symptoms including ptosis (drooping of the upper eyelid), miosis (constricted pupil), and anhidrosis (lack of sweating) on the affected side. One of its key features is the presence of a miotic pupil, often referred to as a Horner's pupil.To differentiate a Horner's pupil from other causes of miosis and to localize the lesion causing Horner's syndrome, pharmacologic testing can be used.a. Hydroxyamphetamine: This can be used to differentiate postganglionic from preganglionic lesions in Horner's syndrome. If dilation occurs after instillation, the lesion is postganglionic. If there's no dilation, the lesion is preganglionic.c. Apraclonidine: This is an alpha-2 adrenergic agonist. In Horner's syndrome, there is denervation hypersensitivity to alpha-adrenergic agents. In patients with acute Horner's syndrome, the instillation of apraclonidine	The correct answer is A, hydroxyamphetamine. Hydroxyamphetamine is a sympathomimetic agent that stimulates the release of norepinephrine from postganglionic sympathetic nerve terminals. In the presence of a Horner syndrome, the norepinephrine release is reduced, and thus the pupil will not dilate in response to hydroxyamphetamine. In contrast, brimonidine and apraclonidine are alpha-2 adrenergic receptor agonists that cause pupillary constriction and can be used to differentiate between pharmacologic dilation and Horner syndrome. Pilocarpine is a cholinergic agonist that constricts the pupil and is used to test for parasympathetic function.	b. Brimonidine	I chose the answer c. Apraclonidine because it is a medication that can be used to confirm the presence of Horner syndrome.Horner syndrome is a condition in which the pupil becomes smaller and the eyelid droops due to a problem with the sympathetic nervous system.Apraclonidine is a medication that can reverse this by causing the pupil to dilate.By instilling apraclonidine in both eyes of a patient with Horner syndrome, the pupil dilation effect of apraclonidine can reverse the anisocoria, which is the difference in size between the two pupils.This is because apraclonidine has a weak α1 effect, which causes the pupil dilator to become responsive again.Hydroxyamphetamin e is used to localize the Horner syndrome to either preganglionic or postganglionic levels, but it is not used to confirm the presence of Horner syndrome.Brimonidine is a relatively pure α2-agonist and has no significant α1-adrenergic effect, so it does

			will cause the affected Horner's pupil to dilate, making it larger or equal in size to the normal pupil. This reversal of anisocoria (where the previously smaller pupil becomes the larger pupil) confirms the diagnosis of Horner's syndrome.d. Pilocarpine: In a very dilute form, pilocarpine can be used to diagnose Horner's syndrome because the denervated iris in Horner's syndrome is supersensitive to cholinergic agents. However, apraclonidine is often preferred due to its quicker onset and clearer results.Given the choices provided:<br>c. Apraclonidine is the drug that can be used to confirm the presence of a Horner pupil.			not produce pupil dilation and is not used to confirm the presence of Horner syndrome.Pilocarpine is a cholinergic medication used to treat dry eye and is not used to confirm the presence of Horner syndrome.
**Disease**	**question**	**standard answer**	**GPT-4**	**GPT-3.5 Turbo**	**Baichuan-13B (Baseline model)**	**ChatZOC (LLM+COD)**
Glaucoma	Patient: Female, 42 years old. Symptoms: Left eye vision decline over six months, worsening recently.<br>Medical History: No diabetes, hypertension for 2+ years, medicated. Tall, thin, with long limbs.<br>Eye History: Right eye blind for 10+ years, left eye high myopia. Family history of poor vision.<br>Examination: Right eye NLP, left eye HM/50cm. Right eye pressure 33mmHg, left 12mmHg. Right eye has corneal changes, large pupil, and	E This patient has absolute stage glaucoma in the right eye and has no visual function, so he should be treated with medication to lower the intraocular pressure conservatively to relieve the glaucoma symptoms, and if the medication is effective in conservative treatment, anti-glaucoma surgery should not be considered for the time being; and there is no light perception in the right eye, so there is no therapeutic value of performing corneal transplantation.	The main manifestation of secondary glaucoma in the patient's right eye is an increase in intraocular pressure (IOP) to 33 mm Hg. Secondary glaucoma is glaucoma due to other ocular diseases or injuries, and this patient's increased IOP in the right eye may be related to lens dislocation. The treatment options described above are explained as follows: A. Glaucoma drainage valve implantation: to control IOP by implanting a drainage valve for refractory glaucoma. B.	Initial treatment for secondary glaucoma in the patient's right eye is usually: E. Topical IOP-lowering medication to control IOP. The IOP in the right eye is markedly elevated (33 mmHg), which is a sign of secondary glaucoma. In managing secondary glaucoma, the primary goal is to lower IOP to minimize damage to ocular structures from intraocular pressure. Topical IOP-lowering medications are part of the initial treatment and are typically used in	E. Topical IOP-lowering agents to control intraocular pressure	E. The patient's right eye absolute stage glaucoma, already aphakic function, so should be carried out medication to reduce intraocular pressure conservative treatment to relieve glaucoma symptoms, if the medication is effective in conservative treatment, do not consider the anti-glaucoma surgery; and the right eye has no light perception, there is no therapeutic value of corneal transplantation.

© Luo MJ et al. *JAMA Ophthalmology*.

	no visible lens; posterior segment unclear. Left eye has clear cornea and anterior chamber, sluggish pupil reflex, no visible lens, and retinal detachment in specific areas.<br>Diagnosis & Plan: Left eye retinal detachment, right eye posterior segment unclear. Needs bilateral B-ultrasound, UBM for lens status. OCT for left eye macular hole. Possible Marfan syndrome; needs cardiac ultrasound, chest X-ray, limb X-rays. Focus on eye-related exams.<br>Marfan Syndrome Risks: High myopia leading to retinal detachment and lens dislocation risks.<br>Treatment: Left eye vitrectomy with lensectomy, internal membrane peeling, gas-liquid exchange, and silicone oil filling. Lens dislocation precludes cataract surgery; limited recovery prospects.<br>The current initial treatment for secondary glaucoma in the patient's right eye is<br>A. Glaucoma drainage valve implantation<br>B. Trabeculectomy<br>C. penetrating corneal transplantation<br>D. Corneal endothelial transplantation<br>E.Topical IOP-lowering medication to control intraocular pressure		Trabeculectomy: traditional open surgery to reduce IOP by decreasing the production of aqueous humor or increasing aqueous humor outflow. C. Penetrating corneal transplantation: for patients with a loss of corneal clarity. D. Endothelial grafting: for the treatment of endothelial dysfunction. E. Localized lowering of IOP. E. Topical IOP-lowering medications to control IOP: medications are used to lower IOP, but medications alone may not be sufficient for secondary glaucoma, especially given that the patient is already blind in one eye. In summary, the first thing that should be tried in this patient is to use medication to lower the IOP in order to stabilize the patient's condition. However, long-term options, especially given the lens dislocation and other problems in the right eye, may require surgical treatment, but the choice of which surgery to perform will depend on the patient's specific condition and the physician's evaluation. Therefore, the current initial treatment for secondary glaucoma in the patient's right eye is E. Topical IOP-lowering medication to control the IOP. However, this is only a temporary treatment, and subsequent surgery may be required.	glaucoma patients to reduce intraocular pressure. Surgical treatments such as glaucoma drainage valve implantation or trabeculectomy may then need to be considered, but the initial treatment is medication to rapidly lower the IOP. Other options are explained as follows: A. Glaucoma drainage valve implantation: this is usually the surgical option considered, but initial treatment usually begins with medication. b. Trabeculectomy: trabeculectomy is a common glaucoma procedure, but it is not usually the initial treatment, but is considered after medication has failed, or when more expedient control of the IOP is needed. c. Penetrating corneal graft: penetrating corneal grafts are usually not be used as the treatment of choice for glaucoma, but rather as a procedure for corneal problems.D. Corneal Endothelial Transplantation: Corneal endothelial transplantation is usually a procedure used to treat dysfunction of the corneal endothelium, and is not directly related to glaucoma treatment. Therefore, in this case, the initial treatment is the use of topical IOP-lowering medications to control IOP.		

Disease	question	standard answer	GPT-4	GPT-3.5 Turbo	Baichuan-13B (Baseline model)	ChatZOC (LLM+COD)
Diabetic Retinopathy	Patient: Male, 66 years old.<br><br>Medical History: Hypertension for 5 years, diabetes for 10 years.<br>Symptoms: Complains of decreased vision in the right eye for 2 weeks.<br>Examination Findings: Right eye vision 0.1, left eye vision 0.8. Both eyes NCTod 11mmHg, os 12mmHg. No significant abnormalities in the anterior segment of both eyes. Hemorrhage in the lower vitreous of the right eye.<br>Diagnosis and Treatment Plan: For a patient with unilateral vitreous hemorrhage, dilated fundus examination of both eyes is essential for a definitive diagnosis and to prevent misdiagnosis in the other eye. B-ultrasound of the affected eye can clarify the retinal condition, including potential retinal or choroidal detachment. Given the patient only has lower vitreous hemorrhage and the upper retina can be partially visualized, bilateral FFA (fundus fluorescein angiography) can be performed for definitive diagnosis and differential diagnosis.<br>Findings Post-Dilation: Right eye's upper retina is flat, scattered with microaneurysms,	B Scattered microangiomas, foci of hemorrhage, and exudates in the retina of both eyes, combined with the history of an elderly male with a 10-year history of diabetes mellitus, suggest that diabetic retinopathy is most likely.	Based on the patient profile given, the patient had a 10-year history of diabetes mellitus. Examination revealed scattered microangiomas, foci of hemorrhage and exudates, which are typical signs of diabetic retinopathy. Diabetic retinopathy is an ocular disease complicated by diabetes mellitus, of which microangiomas are the earliest signs and characteristic lesions. Therefore, the most likely diagnosis is: B. Diabetic retinopathy in both eyes<br>Explanation: Diabetic retinopathy is caused by hyperglycemia that damages the retinal blood vessels, resulting in abnormal neovascularization, hemorrhage, exudation, and microangiomas. The patient's funduscopic findings are consistent with the typical presentation of this lesion.	Based on the patient's history and fundus examination, the most likely diagnosis for this patient is: B. Binocular diabetic retinopathy This is due to the patient's history of hypertension and diabetes mellitus, and the fundus examination reveals hemorrhagic foci, microangiomas, and exudates in the retina of both eyes, which is a common manifestation of diabetic retinopathy. Although the upper retina is flat and ambiguous, other lesion manifestations are consistent with diabetic retinopathy. Explanation of other options: A. Binocular age-related macular degeneration: usually presenting with decreased central vision and changes in the macula that are not consistent with the patient's symptoms and fundus examination. C. Binocular peripapillary retinal veinitis: this condition is usually accompanied by inflammation of the retinal arteries or veins, but is not consistent with a history of diabetes mellitus. D. Binocular retinal lacunae: Lacunae are usually not associated with large hemorrhages and microvascular tumors and are not associated with a history of diabetes mellitus. E. Central retinal	Based on the patient's clinical presentation and examination findings, the most likely diagnosis is B. Diabetic retinopathy of both eyes.<br>EXPLANATION: The patient has a long history of hypertension and diabetes mellitus, which are major risk factors for diabetic retinopathy. In addition, the patient has an accumulation of blood in the vitreous below the right eye, which further aggravates the risk of retinopathy. On fundus examination after pupil dilation, hemorrhagic foci, microangiomas, and exudates were noted to varying degrees in both eyes, changes consistent with the presentation of diabetic retinopathy. Although both eyes had some degree of retinopathy, diabetic retinopathy was chosen as the primary diagnosis because the right eye was sicker.	The patient's diagnosis was most likely diabetic retinopathy in both eyes.<br>EXPLANATION: Based on the patient's history and findings, the patient had a history of hypertensive disease and diabetes mellitus, decreased vision in the right eye, funduscopic examination revealed vitreous hemorrhage in the lower right eye, NCTod11mmHg and os 12mmHg in both eyes, and there were no obvious abnormalities in the anterior visual segments of both eyes, and vitreous hemorrhage in the lower right eye. Dilated pupils of both eyes revealed flat retina with scattered microangiomas, hemorrhagic foci and exudates over the right eye and flat retina with scattered hemorrhagic foci, microangiomas and exudates over the left eye. Diabetes is a systemic disease, so controlling blood sugar is critical. Whole retina photocoagulation is used in the proliferative phase to prevent neovascularization and to degenerate the neovascularization that has formed. Anti-VEGF medication may be considered for macular edema. When complicated by neovascular glaucoma, vitreous cavity injection of anti-VEGF medication

© Luo MJ et al. *JAMA Ophthalmology*.

	hemorrhages, and exudates. Left eye's retina is flat, scattered with hemorrhages, microaneurysms, and exudates.<br>Probable Diagnosis: Based on these findings, the most likely diagnosis is:<br>A. Bilateral age-related macular degeneration<br>B. Bilateral diabetic retinopathy<br>C. Bilateral retinal vasculitis<br>D. Bilateral retinal hole<br>E. Central retinal vein occlusion			vein occlusion: this condition usually presents with macular edema and hard exudates caused by central retinal vein occlusion, which is inconsistent with the patient's symptoms and fundus examination.		followed by drainage valve implantation can be considered for treatment to reduce the risk of intraoperative anterior chamber blood accumulation. When vitreous blood accumulation cannot be self-absorbed and complicates pulling retinal detachment, PPV surgical treatment is required. Therefore, based on the patient's symptoms, signs and retinopathy manifestations, the diagnosis of this patient is most likely diabetic retinopathy in both eyes.
**Disease**	**question**	**standard answer**	**GPT-4**	**GPT-3.5 Turbo**	**Baichuan-13B (Baseline model)**	**ChatZOC (LLM+COD)**
Rare eye diseases	Patient: Female, 3 years old. Presented with “swelling in both eyes since infancy for 3 years.” Eye examination: Vod light chasing, Vos light chasing, pale yellow flat oval swelling measuring 5mmx6mm on the temporal superior bulbar conjunctiva of both eyes, hard in texture, growing about 1mmx0.5mm towards the cornea, no other abnormalities in both eyes. MRI indicates abnormal lipid signals in the temporal conjunctival area of both eyes. History of bilateral preauricular appendage removal surgery.<br>First consideration for this disease is Goldenhar Syndrome. Besides eye symptoms, Goldenhar	A,B,C,D,E The ocular manifestations of Goldenhar syndrome are eyelid defects, corneoconjunctival dermatomas, microphthalmos, amblyopia, and in some cases canthal defects, iris atrophy, cataracts, and choroidal defects.	Goldenhar syndrome, also known as oculo-auriculo-vertebral spectrum (OAVS), is a rare congenital anomaly that does affect the eyes, ears, spine, and several other areas. Based on the description: a yellowish flat oval mass in the superior temporal bulbar conjunctiva of both eyes is most likely a conjunctival dermatomas.Other common manifestations of the eye in Goldenhar syndrome include eyelid defects and microphthalmos. Therefore, based on the above information, for Goldenhar syndrome, the possible manifestations of the eye are:A. Eyelid defects - Correct.Goldenhar	Goldenhar syndrome can lead to a range of eye manifestations, which may include the following options:B. Conjunctival dermatomas: A common manifestation in the eyes of people with Goldenhar syndrome is conjunctival dermatomas, which are abnormal skin or tissue growths on the conjunctiva.C. Microphthalmos: Goldenhar syndrome can also lead to microphthalmos (tiny eyes) or ocular hypoplasia, which This may cause abnormalities in the size and shape of the eye. d. Corneal dermatomas: Although not as common as conjunctival dermatomas, dermatomas of the cornea can also be	B. Conjunctival dermatomas	1. In this disease, the ocular manifestations may be: (Multiple choice) A. Eyelid defect B. Conjunctival dermatoid tumor C. Microphthalmia D. Keratodermatoid tumor E. Amblyopia Explanation: According to the child's symptoms and examination results, the ocular manifestations are 5 mm x 6 mm yellowish flat oval mass in the superior temporal conjunctiva of both eyes, hard, and growing toward the cornea of about 1 mm x 0.5 mm, which is in line with the manifestations of conjunctival dermatoid tumor. It was consistent with a conjunctival dermatoid tumor.

© Luo MJ et al. *JAMA Ophthalmology*.

	Syndrome also manifests with ear deformities (microtia or preauricular appendages), facial asymmetry, spinal deformities, cleft lip and palate, hearing impairment, and congenital heart disease.<br>Question: Based on the above information, possible eye manifestations of this disease include:<br>A. Eyelid defect<br>B. Conjunctival dermoid<br>C. Microphthalmos<br>D. Corneal dermoid<br>E. Amblyopia		syndrome may be accompanied by congenital defects of the eyelids.B. Conjunctival dermatofibrosarcoma - Correct.B. Conjunctival dermatofibrosarcoma - Correct.B. Conjunctival dermatofibrosarcoma - Correct. The description of a mass in the superior temporal bulbar conjunctiva of both eyes is consistent with conjunctival dermatomas.C. Microphthalmia - Correct. Microphthalmia (microphthalmia) is a common ocular manifestation of Goldenhar syndrome.D. Keratoconjunctival dermatomas - Incorrect. The swelling mentioned in the description grows on the conjunctiva, not the cornea.E. Amblyopia - Not mentioned in the description given or in the common manifestations of Goldenhar syndrome, so this option cannot be determined to be correct. However, given the possible ocular abnormalities in patients with Goldenhar syndrome, amblyopia may be a complication. In summary, the correct answers are A, B, and C.	present in Goldenhar syndrome. a. Eyelid defects: Eyelid defects are not usually a direct ocular manifestation of Goldenhar syndrome, although they may be associated with other disorders. e. Amblyopia: Individuals with Goldenhar syndrome can have ocular malformations. can have ocular malformations that may affect visual development, but amblyopia is usually a visual problem rather than a direct ocular manifestation. It is important to note that Goldenhar syndrome is a multisystem disorder and can be associated with problems in other organs and systems in addition to ocular manifestations. Therefore, comprehensive evaluation and treatment of patients with Goldenhar syndrome is very important.		
**Disease**	**question**	**standard answer**	**GPT-4**	**GPT-3.5 Turbo**	**Baichuan-13B (Baseline model)**	**ChatZOC (LLM+COD)**
Acute anterior uveitis	Patient: Young male, experiencing redness, pain, photophobia, and decreased vision in the	C Typical symptoms of acute anterior uveitis: pain, photophobia, and may be accompanied by	Patients present with typical symptoms of acute anterior uveitis such as redness and pain,	Based on the patient's description, the most appropriate treatment option is: C. Dilating the	Based on the patient's symptoms and ocular findings, a preliminary diagnosis of uveitis in the	This question examines the principles of treatment for acute anterior uveitis. Typical symptoms of acute

© Luo MJ et al. *JAMA Ophthalmology*.

	right eye for 4 days. Eye examination: Right eye vision 0.6, no improvement with correction, conjunctival ciliary congestion ＋, dust-like KP(++)Tyn(+), aqueous cells(++), round pupil with a diameter of about 2.5mm, clear lens, no vitreous opacities, and normal fundus. Left eye vision 1.0, no congestion, clear cornea, KF), 3.5mm pupil, clear lens, and normal fundus. Normal eye pressure.<br>Question: Based on the current condition of the patient, which group of drugs is the first choice for treatment?<br>A. Pupil constriction, corticosteroid eye drops<br>B. Pupil dilation, corticosteroid eye drops, intravenous corticosteroid infusion<br>C. Pupil dilation, corticosteroid eye drops<br>D. Pupil constriction, oral corticosteroids<br>E. Pupil dilation, corticosteroid eye drops, oral corticosteroids	decreased visual acuity. Typical signs are inflammatory manifestations in the anterior segment of the eye: ciliary congestion, dusty or goopy KP, visible aqueous humor proteins, aqueous humor cells, and pupillary dilation. The posterior segment of the eye was normal and the intraocular pressure was normal, so the first consideration was "acute anterior uveitis". The treatment principle of acute anterior uveitis is: dilate the pupil, at the same time, topical glucocorticoid anti-inflammatory. If the inflammation is severe, glucocorticoids can be added to the whole body. However, the patient's anterior uveitis was not severe enough to require systemic glucocorticoids.	photophobia, decreased visual acuity, conjunctival congestion, corneal KP (dusty deposits), and atrial hydrocytes. The primary goals of treatment for anterior uveitis are to control inflammation, reduce symptoms, and prevent complications. Let's analyze the options:<br>A. Pupil constriction, glucocorticoid punctures: Pupil constriction medications are usually used in acute anterior uveitis to reduce light pain and prevent pupillary adhesions. Glucocorticoid punctures are used to control inflammation of the cornea and uvea.B. Pupil dilation, glucocorticoid punctures, and glucocorticoid IV: This combination is more appropriate for patients with severe uveitis or in the presence of other complications.C. Pupil dilation, glucocorticoid punctures: Pupil-dilating medications are not the first choice in this case.D. Pupil dilation, glucocorticoid orally: For generalized anterior uveitis, glucocorticosteroids taken orally may be overtreatment.E. Pupil dilation, glucocorticoid punctures, oral glucocorticoid: same as D. This combination may be overtreatment in mild to moderate anterior uveitis. Therefore, based on the	pupil and glucocorticoid eye drops The patient's right eye presents with redness, pain, and decreased visual acuity with conjunctival congestion, dusty KP, and increased atrial aqueous humor cells, which are indicative of possible iridocyclitis or anterior chamber keratitis. The usual treatment includes the use of pupil-dilating medications to reduce iridocyclitis spasm and inflammation, and glucocorticoid eye drops to reduce ocular inflammation. Therefore, option C is the preferred treatment option. In cases of significant symptoms or severe disease, physicians may consider adding oral glucocorticoids (Option D), but eye-dotting is generally the preferred treatment.	right eye is made. In this case, the preferred pharmacologic regimen is A. Pupil constriction with glucocorticoid punctures. This is because glucocorticoids reduce the inflammatory response, while pupil-contracting medications such as Trichostatin help reduce the discomfort associated with pupil dilation. Also, glucocorticosteroids can be used for intraocular injections or eye punctures to act directly on the affected area. Other options such as pupil dilation and oral glucocorticoids are not indicated for the treatment of this condition.	anterior uveitis include pain, photophobia, and may be accompanied by decreased visual acuity. Typical signs are inflammatory manifestations in the anterior segment of the eye: ciliary congestion, dusty or goopy KP, visible aqueous humor proteins, aqueous humor cells, and pupillary dilation. The posterior segment of the eye was normal and the intraocular pressure was normal, so the first consideration was "acute anterior uveitis". The treatment principle of acute anterior uveitis is: dilate the pupil, at the same time, local glucocorticoid anti-inflammatory. If the inflammation is severe, glucocorticoids can be added to the whole body. However, the patient's inflammation in the anterior segment of the eye is not severe, and systemic glucocorticoids are not needed. Based on the patient's current condition, the preferred pharmacologic treatment is dilation of the pupil and glucocorticoid punctures. So the answer is C.

			above information and the patient's presentation, the preferred pharmacologic regimen should be A. pupil dilation, glucocorticoid spotting.			

The question-answer pairs were originally in Chinese when used to test the models, and they were translated into English using Google translate (www.translate.google.com).

© Luo MJ et al. *JAMA Ophthalmology*.
