# Supplementary material for: Development and Evaluation of a Retrieval-Augmented Large Language Model Framework for Ophthalmology
Source: JAMA Ophthalmol. 2024 Jul 18;142(9):798–805. doi: 10.1001/jamaophthalmol.2024.2513 (PMC11258636; doi:10.1001/jamaophthalmol.2024.2513)
Supplement: Supplement 2. — Data Sharing Statement. [file jamaophthalmol-e242513-s002.pdf]

Data Sharing Statement

Luo. Development and Evaluation of a Retrieval-Augmented Large Language Model Framework for Ophthalmology. *JAMA Ophthalmol*. Published July 18, 2024.
doi:10.1001/jamaophthalmol.2024.2513

Data
Data available: Yes
Data types: Data (not involving human participants)
How to access data: luomingjie@gzzoc.com
When available: With publication

Supporting Documents
Document types: None

Additional Information
Who can access the data: researchers whose proposed use of the data has been approved
Types of analyses: for any purpose
Mechanisms of data availability: after approval of a proposal
